# A nonsense *TMEM43* variant leads to disruption of connexin-linked function and autosomal dominant auditory neuropathy spectrum disorder

**DOI:** 10.1073/pnas.2019681118

**Published:** 2021-05-28

**Authors:** Minwoo Wendy Jang, Doo-Yi Oh, Eunyoung Yi, Xuezhong Liu, Jie Ling, Nayoung Kim, Kushal Sharma, Tai Young Kim, Seungmin Lee, Ah-Reum Kim, Min Young Kim, Min-A Kim, Mingyu Lee, Jin-Hee Han, Jae Joon Han, Hye-Rim Park, Bong Jik Kim, Sang-Yeon Lee, Dong Ho Woo, Jayoung Oh, Soo-Jin Oh, Tingting Du, Ja-Won Koo, Seung-Ha Oh, Hyun-Woo Shin, Moon-Woo Seong, Kyu-Yup Lee, Un-Kyung Kim, Jung Bum Shin, Shushan Sang, Xinzhang Cai, Lingyun Mei, Chufeng He, Susan H. Blanton, Zheng-Yi Chen, Hongsheng Chen, Xianlin Liu, Aida Nourbakhsh, Zaohua Huang, Kwon-Woo Kang, Woong-Yang Park, Yong Feng, C. Justin Lee, Byung Yoon Choi

**Affiliations:** ^a^KU-KIST Graduate School of Converging Science and Technology, Korea University, Seoul 02841, Republic of Korea;; ^b^Center for Cognition and Sociality, Institute for Basic Science, Daejeon 34141, Republic of Korea;; ^c^Department of Otorhinolaryngology, Seoul National University Bundang Hospital, Seongnam 13620, Republic of Korea;; ^d^College of Pharmacy and Natural Medicine Research Institute, Mokpo National University, Muan 58554, Republic of Korea;; ^e^Department of Otolaryngology, University of Miami Miller School of Medicine, Miami, FL 33136;; ^f^Dr. John T. Macdonald Foundation Department of Human Genetics, Hussman Institute for Human Genomics, University of Miami Miller School of Medicine, Miami, FL 33136;; ^g^Department of Otolaryngology, Xiangya Hospital, Central South University, Changsha, Hunan 410008, China;; ^h^Institute of Molecular Precision Medicine, Xiangya Hospital, Central South University, Changsha, Hunan 410008, China;; ^i^Samsung Medical Center, Samsung Genome Institute, Seoul 06351, Republic of Korea;; ^j^Department of Biology, College of Natural Sciences, Kyungpook National University, Daegu 41566, Republic of Korea;; ^k^School of Life Sciences, KNU Creative BioResearch Group (BK21 plus project), Kyungpook National University, Daegu 41566, Republic of Korea;; ^l^Department of Biomedical Sciences, Seoul National University Graduate School, Seoul 03080, Republic of Korea;; ^m^Department of Otorhinolaryngology, Seoul National University Hospital, Seoul 03080, Republic of Korea;; ^n^Research Center for Animal Model, Jeonbuk Department of Inhalation Research, Korea Institute of Toxicology, Jeongeup 56212, Republic of Korea;; ^o^Convergence Research Center for Diagnosis, Treatment and Care System of Dementia, Korea Institute of Science and Technology, Seoul 02792, Republic of Korea;; ^p^Department of Neuroscience, University of Virginia, Charlottesville, VA 22908;; ^q^Department of Laboratory Medicine, Seoul National University Hospital, Seoul 03080, Republic of Korea;; ^r^Department of Otorhinolaryngology-Head and Neck Surgery, Kyungpook National University Hospital, Daegu 41944, Republic of Korea;; ^s^Department of Otology and Laryngology, Harvard Medical School and Eaton-Peabody Laboratory, Boston, MA 02114;; ^t^Department of Otolaryngology, University of South China Affiliated Changsha Central Hospital, Changsha, Hunan 410004, China

**Keywords:** auditory neuropathy spectrum disorder, cochlea, glia-like supporting cells, connexins

## Abstract

Genes that are primarily expressed in cochlear glia-like supporting cells (GLSs) have not been clearly associated with progressive deafness. Herein, we present a deafness locus mapped to chromosome 3p25.1 and an auditory neuropathy spectrum disorder (ANSD) gene, *TMEM43*, mainly expressed in GLSs. We identify p.(Arg372Ter) of *TMEM43* by linkage analysis and exome sequencing in two large Asian families segregating ANSD, which is characterized by inability to discriminate speech despite preserved sensitivity to sound. The knock-in mouse with the p.(Arg372Ter) variant recapitulates a progressive hearing loss with histological abnormalities in GLSs. Mechanistically, TMEM43 interacts with the Connexin26 and Connexin30 gap junction channels, disrupting the passive conductance current in GLSs in a dominant-negative fashion when the p.(Arg372Ter) variant is introduced. Based on these mechanistic insights, cochlear implant was performed on three subjects, and speech discrimination was successfully restored. Our study highlights a pathological role of cochlear GLSs by identifying a deafness gene and its causal relationship with ANSD.

## Significance

Auditory neuropathy spectrum disorder (ANSD) is a confounding auditory disease in which the subjects respond to sound but have difficulties in speech discrimination. Herein, we examined two Asian families with hereditary late-age–onset ANSD. By linkage analysis and exome sequencing, we identified the TMEM43-p.(Arg372Ter) variant as the etiology of the disease. To examine the mechanism of TMEM43 on ANSD, we generated a knock-in mouse with the p.(Arg372Ter) variant that recapitulated the progressive hearing loss phenotype of the two families. We discovered that variation in TMEM43 impairs the connexin-linked function of mediating passive conductance current in cochlear glial cells. Based on the pathology involving cochlear glial cells, we performed cochlear implant on the human patients, and their speech discrimination ability was restored.

Auditory neuropathy spectrum disorder (ANSD) is defined as an inability in speech discrimination despite preserved sensitivity to sound (1). Clinically, ANSD has been characterized by the presence of otoacoustic emission (OAE) and/or cochlear microphonics (CM) and the concurrent absence of averaged auditory brainstem responses (ABR) or presence of abnormal ABR (2, 3). The presence of OAE and/or CM is indicative of normal cochlear outer hair cell (OHC) activity, whereas the abnormal ABR is indicative of disrupted auditory nerve (AN) activity (2). Up to now, research has suggested that ANSD is caused by a malfunctioning of the inner hair cells (IHC), the synapse between the IHCs and AN, or AN itself such as demyelination or desynchronization (4–6). However, in the organ of Corti of inner ear, glia-like supporting cells (GLSs) comprise major cell types in addition to OHCs and IHCs. GLSs are defined as glia-like cells due to a presence of typical glia markers such as GFAP and GLAST (7). While hair cells play critical roles in mechanoreception and synaptic transmission by converting the acoustic energy into electrochemical signals that are relayed to the brainstem (7), GLSs reside adjacent to hair cells and play critical roles in development and maintenance of auditory system (7–10). Developing mammalian cochlea can be categorized in two anatomical regions as greater epithelial ridge (GER) and lesser epithelial ridge (LER) (11). The GER refers to the area medial to pillar cells including IHCs and inner GLSs of Kolliker’s organ such as inner phalangeal cells and border cells (12, 13). The LER spans the area radial to pillar cells, consisting of OHCs and outer GLSs such as Hensen’s cells and Deiters’ cells (12). The GLSs in Kolliker’s organ have been studied to generate ATP- and TMEM16A-dependent spontaneous activity that depolarizes IHCs (10, 11, 13), generate spontaneous Ca^2+^ signaling for OHC refinement (14), and spontaneously regenerate hair cell in the neonatal mouse cochlea (9, 15, 16). Despite their essential function at prehearing stages, the precise role of GLSs in hearing or speech discrimination, especially their contribution to late-onset ANSD, remains unknown.

The GLSs are physically coupled to each other by gap junctions (7). Gap junctions consist of two hemi channels, which are encoded by connexin genes, that meet at the plasma membrane of adjacent cells to achieve cell coupling. The connexins provide a pathway for rapid removal of ions from the region of the hair cells during sound transduction (17) by recycling and regulating intracellular K^+^ and maintaining pH homeostasis (18–20). Connexin26 (Cx26, GJB2) and Connexin30 (Cx30, GJB6) are the two predominantly expressed connexins in the GLSs of mammalian cochlea (21) and also the major deafness genes known to induce high incidence of nonsyndromic hearing loss (21–24). Variants in connexins break the endocochlear potential of the inner ear and lead to hearing loss (21). Although a pathological role of connexin variants on ANSD has been suggested (25), a combination of heterozygous phenotypes, diverse Cx26 and Cx30 variants (21), and various interacting proteins (26, 27) make it difficult to determine the molecular and cellular mechanism of connexin-related ANSD. Moreover, none of the known interacting proteins for connexins has been associated with ANSD.

In this study, we identified two Asian families with hereditary late-onset ANSD with progressive hearing loss. By linkage analysis and exome sequencing, we determined TMEM43-p.(Arg372Ter) variant as the origin of the disease. In order to examine the role of TMEM43, we generated a knock-in (KI) mouse with p.(Arg372Ter) variant which recapitulated a progressive hearing loss with ANSD phenotypes. Ex vivo and in vitro studies further demonstrated that TMEM43 physically interacts with Cx26 and Cx30 in the GLSs. When the p.(Arg372Ter) variant was introduced, passive conductance current in GLSs was disturbed with histological abnormalities in GLSs. Based on the mechanistic insights, cochlear implant (CI) was performed on patients with p.(Arg372Ter) variant, and their speech discrimination was successfully restored. Our study identifies an interacting protein of connexins in cochlea and introduces a role of GLSs in late-onset ANSD.

## Results

### *TMEM43* Is a Novel Deafness Gene.

**Fig. 1.**
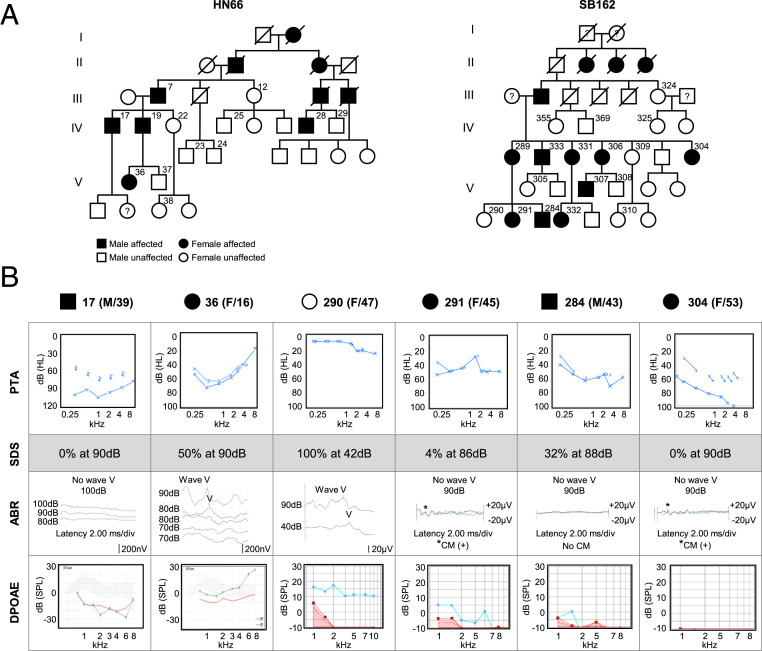
Pedigree and audiological assessment of family HN66 and SB162. (*A*) Pedigree of Chinese family HN66 and a Korean family SB162 family segregates ANSD in an autosomal dominant fashion. Open and filled symbols indicate unaffected and affected statuses, respectively. Square and circle symbols indicate male and female, respectively. Subject numbers are superscripted. (*B*) PTA, SDS, ABR, and DPOAE of six subjects from HN66 and SB162. The behavioral hearing threshold of each subject can be identified in the PTA results, and ABR tests identify the electrophysiological hearing threshold where wave V disappears. In DPOAE, red line indicates level of noise, and blue or green line indicates level of response. The gray shading in #17 and #36 indicates a normative region. The control subject #290 shows 40 dB or lower ABR threshold. #36, #291, and #304 show no response to 90 dB stimulus (#291 and #304) or 80 dB of hearing threshold as shown by the overlapping wave V to the threshold stimulus of the same magnitude in the two repetitive measurements (#36) in ABR, while displaying either positive DPOAE responses (#36 and #291) or presence of CM (asterisk) (#304). The subject #36 showed disordered waveform differentiation in ABR responses and significant elevation of ABR threshold up to 80 dB, with preserved DPOAE response. #17 and #284 who do not elicit any ABR response show none or very subtle DPOAE response which does not suffice for diagnosis for ANSD. Subjects #17 and #36 from Chinese family HN66 share the same ABR scale, and subjects #290, #291, #284, and #304 from Korean family SB162 share the same ABR scale.

We identified two large five-generation pedigrees of Chinese Han family (HN66) and Korean family (SB162), nonconsanguineously segregating the late-onset progressive ANSD in an autosomal dominant fashion (Fig. 1*A* and *SI Appendix*, Fig. S1*A*). Unlike the normal subject (#290) in SB162, the affected subjects (#17, #291, #284, and #304) from families HN66 and SB162 displayed elevated pure-tone audiogram (PTA) thresholds and disproportionately lower speech discrimination score (SDS) for the PTA thresholds. Importantly, complete absence of ABR was noted from these affected subjects, despite the presence of either distortion-product OAE (DPOAE) or CM (Fig. 1*B*), which are classic signs of ANSD. CM measurement was not performed from subjects #17 and #36. In subjects #374 and #376 from SB162, elevation of low-frequency pure-tone hearing thresholds, which was not subjectively detectable and not accompanied by decrease in speech discrimination, began slowly from the age of 10 (*SI Appendix*, Fig. S1*A*), and the typical ASND symptoms appeared from the middle of second decade as shown in subject #36 from HN66 (a 16-y-old female) (Fig. 1*B*). She showed a significant elevation of ABR threshold as high as 80 dB, while DPOAE response was completely preserved (Fig. 1*B*). This suggests that phenotype for #36 can be interpreted as the stage of transition to canonical ANSD. In addition, we measured PTA in time series from three individuals (#284, #374, and #376) and found a time-dependent progression of hearing loss (*SI Appendix*, Fig. S1*A*). Given significant aggravation of hearing over 15 y in #284 from SB162, we concluded that the progression of ANSD appears to be particularly pronounced in the 30s (*SI Appendix*, Fig. S1*A*).

**Fig. 2.**
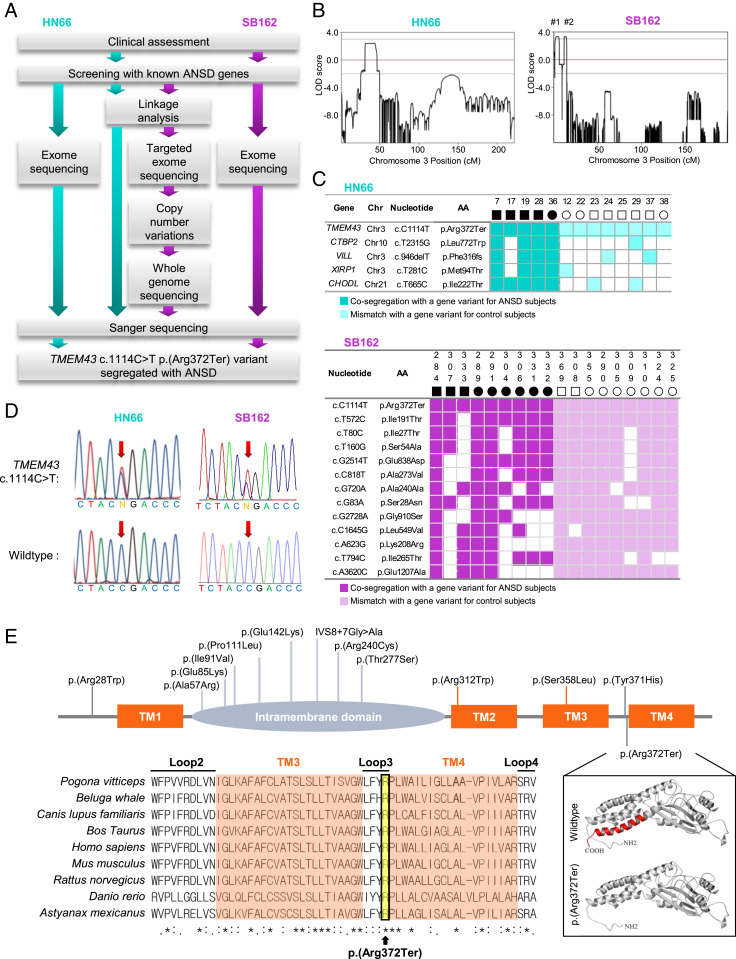
A p.(Arg372Ter) variant of *TMEM43* is the causative variant of ANSD in family HN66 and SB162. (*A*) Overall workflow of variant detection in HN66 and SB162. (*B*) Merlin multipoint linkage analysis demonstrates LOD scores greater than 3.0 on chromosome 3p25.1. Region #1 (Chr. 3: 1,946,000 to 5,956,000), region #2 (Chr. 3:11,883,000 to 14,502,000). (*C*) Genetic candidates of the ANSD after exome sequencing of genomic DNA from three subjects (#22, #28, and #36) in HN66 and four subjects (#289, #290, #291, and #284) in SB162. Subsequent segregation study with additional subjects to identify *TMEM43* variant in HN66 and SB162 with full-match was performed. Cosegregation with a gene variant for ANSD subjects in HN66 and SB162 are indicated as cyan and purple box, respectively. Mismatch with a gene variant for control subjects are lightly colored. Chr; chromosome, AA; amino acid. (*D*) Representative DNA sequence chromatograms of c.1114C > T variant in *TMEM43*. Red arrow signifies allele c.1114C. (*E*) The variant spectrum of *TMEM43* associated with ARVC and Emery–Dreifuss muscular dystrophy 7 (EDMD7) reported in human gene mutation database (HGMD) (*Top*) and the variants identified in the present study [*Bottom*, p.(Arg372Ter)]. The conservative amino acid sequences of TMEM43 are shown below. Asterisks, identical amino acids; orange box, TM domains; yellow box, p.(Arg372Ter). A protein structure modeling of WT human TMEM43 and the variant TMEM43 lacking TM4 (red) is shown. TM, transmembrane domain.

A stepwise genetic analysis was carried out to identify the pathogenic variants of families HN66 and SB162, separately (Fig. 2*A*). To identify the chromosomal locus of ANSD in the two families, we conducted a whole-genome linkage-scan on nine subjects (five affected and four unaffected) in HN66 and 18 subjects (10 affected and 8 unaffected) in SB162 (*SI Appendix*, Fig. S1*B*). As a result, we identified a candidate region on chromosome (Chr) 3: 13,165,401 to 22,769,511 with the highest parametric $\log_{10}Odds$ (LOD) value in both families. We calculated LOD of 2.4 across the entire chromosomes in HN66 (Fig. 2 *B*, *Left*) and LOD of greater 3.0 in SB162 (Fig. 2 *B*, *Right*). In particular, we found two regions (Region #1: Chr 3: 1,946,000 to 5,956,000; Region #2: Chr 3: 11,883,000 to 14,502,000) in the chromosome 3p25.1 with a genetic length of 6.7 and 3 cM, in SB162 (Fig. 2 *B*, *Right* and *SI Appendix*, Fig. S2 *A*, *Inset*). Given the failure to show any link with the previously reported ANSD genes such as *OTOF* (NM_194248, 2p23.3) (28), *DIAPH3* (NM_001042517, 13q21.2) (29), *SLC17A8* (NM_139319, 12q23.1) (30), *AIFM1* (NM_004208, Xq26.1) (31), *OPA1* (NM_130837, 3q29) (32), and an ANSD gene reported by us, *ATP1A3* (NM_152296.4, 19q13.2) (33), we suspected an involvement of another gene.

To narrow down another gene, we employed various advanced genomic sequencing techniques to evaluate the affected families. Although the identification of a causative gene for ANSD from these two families was done independently and blindly in China and South Korea, the linkage interval shared by the two families narrowed down to Chr 3: 13,165,401 to 14,502,000. Exome sequencing was first performed from two affected subjects (#36 and #28) and one unaffected subject (#22), yielding five candidate variants cosegregating with the phenotype (*XIRP1*, *CTBP2*, *CHODL*, *TMEM43*, and *VILL*) (*SI Appendix*, Table S1). Next, we performed detailed Sanger sequencing of these variants from 10 additional subjects in HN66 and zeroed in on only one variant [p.(Arg372Ter) from *TMEM43*] which cosegregated with the ANSD phenotype (Fig. 2 *C* and *D*). This variant fell within the linkage interval of HN66 (Chr 3: 13,165,401 to 22,769,511). For SB162, we employed two diagnostic pipelines in a parallel fashion (Fig. 2*A*). First, we performed targeted exome sequencing of the two regions obtained from the linkage analysis among four subjects (#284, #289, #290, and #291), leading to identification of two nonsynonymous variants [*TMEM43*-p.(Arg372Ter) and *FBLN2*-p.(Asp851His)] in Region #2 that cosegregated with the ANSD phenotype (*SI Appendix*, Table S2, *Top*). Of the two variants, we excluded *FBLN2*-p.(Asp851His), based on its high occurrence (0.4%) among the healthy Korean population in two independent databases, Korean Reference Genome Database (KRGDB) (*n* = 1722) and in-house Samsung Genome Institute control subjects database (*n* = 400), zeroing in on p.(Arg372Ter) from *TMEM43* (NM_024334) as the only candidate. No convincing copy number variations, such as a large genomic deletion and duplication, were detected within the locus (*SI Appendix*, Fig. S2*B*). To exclude noncoding causative variants, we also performed whole genome sequencing of the linkage interval from eight subjects (#284, #289, #290, #304, #307, #309, #324, and #332). The filtering step left us with one nonsynonymous variant [TMEM43-p.(Arg372Ter)] and eighteen noncoding region variants that cosegregated with the deaf phenotype within the linkage interval (*SI Appendix*, Table S3). We excluded all of the 18 variants, based on their high occurrence (>0.4%) among the two aforementioned Korean databases from normal controls and detection of these variants among unaffected subjects or normal controls (*n* = 65) by Sanger sequencing, leaving p.(Arg372Ter) from *TMEM43* (NM_024334) as the only candidate (*SI Appendix*, Table S3). Separately from the first pipeline and independently of linkage analysis, we performed exome sequencing from the same four subjects (#284, #289, #290, and #291), identifying 20 possible candidate variants through an extensive filtering process in SB162 (*SI Appendix*, Table S2, *Bottom*). We performed detailed Sanger sequencing of these variants from 14 additional subjects in the SB162 and excluded 19 variants from the potential candidates again leaving only one variant p.(Arg372Ter) from *TMEM43* as a candidate (Fig. 2 *C* and *D* and *SI Appendix*, Fig. S1*C*). Taken together, we narrowed down to *TMEM43*- p.(Arg372Ter) as the potential causative gene for ANSD in two families, HN66 and SB162.

Next, we closely examined the amino acid sequence of TMEM43 for potential pathogenicity. Previously, p.(Ser358Leu) in *TMEM43* has been shown to cause familial arrhythmogenic right ventricular cardiomyopathy (ARVC) (34, 35). Along with the variant spectrum of *TMEM43* associated with the heart diseases reported in the human gene mutation database (Fig. 2 *E*, *Top*), the arginine at position 372 as well as amino acids in the transmembrane domain (TM) and loop regions of TMEM43 are highly conserved across organisms (Fig. 2 *E*, *Bottom* and *SI Appendix*, Fig. S3). A protein structure modeling of human TMEM43 showed that p.(Arg372Ter), which was expected to introduce a premature stop codon, caused a truncation of the last 29 amino acids of the TMEM43 protein. (Fig. 2 *E*, *Inset*). This variant was predicted to be pathogenic, according to the guideline for the interpretation of classifying pathogenic variants (PS3, PM2, PP1_Strong, and PS4_Supporting) (36, 37) and high Combined Annotation-Dependent Depletion score of 45 (38). However, the ANSD subject of HN66 and SB162 displayed symptoms of neither arrhythmia nor any other heart abnormalities. Conversely, hearing loss has not been reported in *TMEM43-*p.(Ser358Leu) ARVC patients (34, 35). This suggests that p.(Ser358Leu) and p.(Arg372Ter) of *TMEM43* exert a completely different pathophysiological mechanism, leading to pleiotropy of this gene.

### TMEM43 Is Expressed in GLSs.

**Fig. 3.**
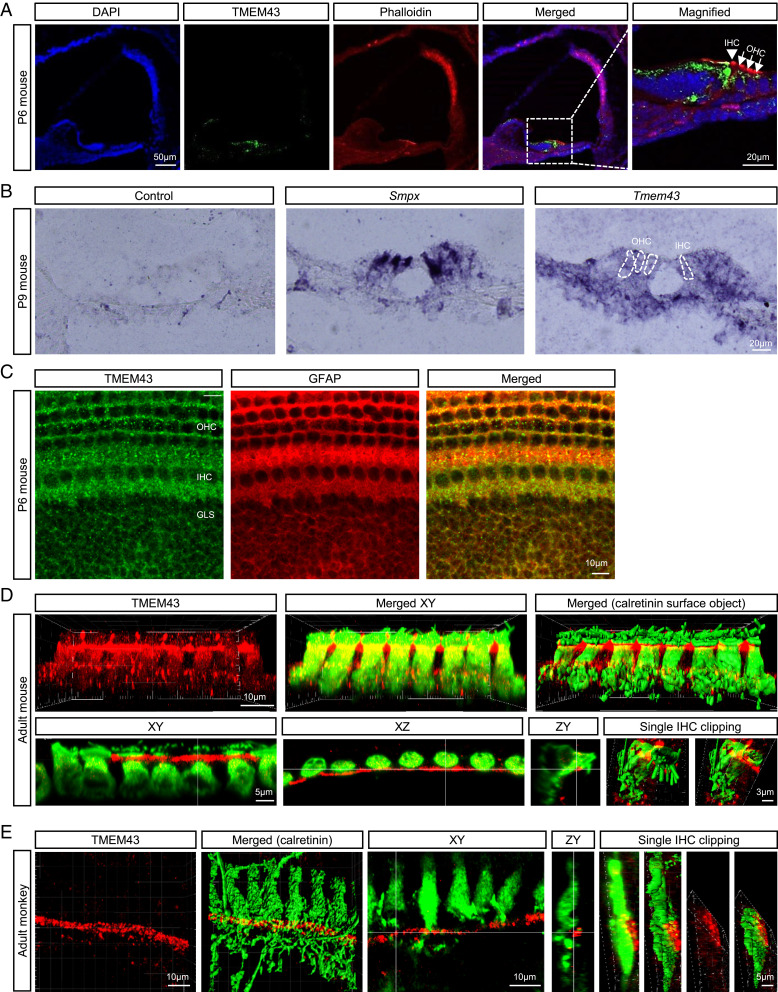
TMEM43 is expressed in the GLSs of mammalian cochlea. (*A*) Frozen-section images of the mouse cochlea at P6 immunolabeled by anti-TMEM43 (green) and counterstained by phalloidin (red). TMEM43 immunoreactivity is detected mainly at the organ of Corti, and the magnified image shows TMEM43 immunoreactivity at the supporting cells of the organ of Corti. Arrow head, IHCs; arrows, OHCs. (*B*) In situ hybridization with a control probe, hair cell marker *Smpx* probe, and *Tmem43* probe showing *Tmem43* mRNA expression in the Organ of Corti. The dotted lines indicate OHCs and IHCs. (*C*) Immunostained cochlea tissue with TMEM43 and glial marker GFAP. (*D* and *E*) Imaris-reconstructed images of cochlea display TMEM43 expression (red) in GLSs that does not colocalize with hair cells (green) in adult mouse (*D*) nor adult monkey (*E*). XY, XZ, and ZY section views and surface object clipping images of a single IHC show that TMEM43 immunoreactivity is found primarily in the apical potion of inner border cell, juxtaposing the upper lateral membrane of calretinin-positive IHC.

To investigate the molecular and cellular mechanisms of TMEM43-associated ANSD, we took a reverse translational approach to phenocopy the genetic disposition in mouse model. The cochlear expression of TMEM43 was firstly confirmed by RT-PCR (*SI Appendix*, Fig. S4*A*). TMEM43 was expressed throughout the mouse body including cochlea, heart, eye, brain, and kidney (*SI Appendix*, Fig. S4*A*). To specify TMEM43 expression in the cochlea tissue, we performed immunostaining using anti-TMEM43 antibody. The expression of TMEM43 protein was evaluated at various time points along the postnatal development of hearing in the mouse cochlea (*SI Appendix*, Fig. S4 *B* and *C*). The previous studies have reported that TMEM43 is localized predominantly to the inner nuclear membrane in multiple noncardiac cell types and also shows some expression outside the nucleus, including the endoplasmic reticulum (35, 39). In the case of ex vivo cochlea, TMEM43 was mainly expressed in the organ of Corti, along the entire cochlear length, at postnatal day 4 (P4) through P20 (Fig. 3*A* and *SI Appendix*, Fig. S4 *B* and *C*). At P20, the expression became more restricted to the apical membrane of the inner border cells and the cell junctions of the inner sulcus cells (*SI Appendix*, Fig. S4*B*). Throughout the early developmental period, up to P20, the TMEM43 expression was found mainly at inner GLSs of Kolliker’s organ, while hair cell expression is comparably sparse, at both protein and messenger RNA (mRNA) levels (Fig. 3 *A* and *B*, *SI Appendix*, Fig. S4 *B* and *C*, and Movies S1 and S2). TMEM43 expression in cochlear glial cell was confirmed again by coexpression with glia-specific GFAP signal (Fig. 3*C*). Adult mice at 1, 2, and 4 mo still maintained TMEM43 protein expression in GLSs (Fig. 3*D* and *SI Appendix*, Fig. S6*C*). This glia-specific TMEM43 expression was recapitulated in adult primates corresponding to age 40's in humans, showing expression in the junctional area between the inner supporting cells and IHCs, without overlapping with calretinin staining (Fig. 3*E*). The specificity of the antibody was confirmed via a significant reduction of immunoreactivity from the cultured mouse organ of Corti when infected with lentivirus carrying *Tmem43*-short hairpin RNA (shRNA) (*SI Appendix*, Fig. S5 *A* and *B*). These results strongly suggest that TMEM43 may play a critical role in cochlear GLSs.

### Localization of TMEM43 in the Plasma Membrane and Loss of Protein Stability in *TMEM43*-p.(Arg372Ter).

**Fig. 4.**
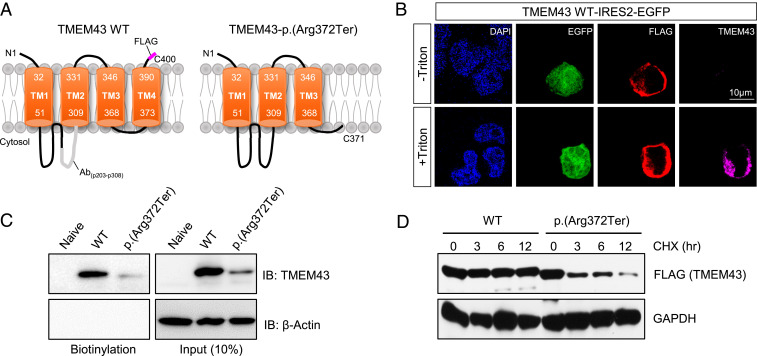
TMEM43-p.(Arg372Ter) variant shows decreased cell surface expression with reduced protein stability. (*A*) Membrane topology of TMEM43 WT and p.(Arg372Ter). Antibody epitope of TMEM43 is indicated as Ab, and FLAG is tagged at C-terminal. (*B*) Immunostaining result of TMEM43 expressing HEK293T cells with and without cell permeabilization. Cells were double stained with FLAG and TMEM43 antibody. (*C*) Cell surface biotinylation assay in TMEM43 WT and p.(Arg372Ter)-transfected HEK293T cells blotted with TMEM43 antibody. (*D*) Cycloheximide chase (CHX) assay with FLAG antibody. Band sizes for TMEM43 WT and TMEM43-p.(Arg372Ter) are 44 kD and 41 kD each.

Based on hydrophobicity analysis, the secondary structure of TMEM43 was predicted to have four TMs and one intramembrane domain with its N- and C-terminal domains located at extracellular space (*SI Appendix*, Fig. S3*A* and Fig. 4*A*) (34). Bioinformatics revealed that TMEM43 was phylogenetically close among many species (*SI Appendix*, Fig. S3*B*), and the four TMs were highly conserved among different species (*SI Appendix*, Fig. S3*C*). We verified the predicted TMEM43 topology through immunocytochemistry with or without cell permeabilization. FLAG-tagged TMEM43 was transfected to human embryonic kidney (HEK) 293T cells and double stained with anti-FLAG and anti-TMEM43 with epitope targeting p203-p308 at intracellular loop1 (Fig. 4*A*). We found that TMEM43 immunoreactivity was positive only with cell permeabilization, indicating that the loop1 of TMEM43 resides at the intracellular space (Fig. 4*B*). In addition, a positive FLAG signal without cell permeabilization was detected (Fig. 4*B*), indicative of membrane expression of TMEM43 and confirming the topology. To verify the cell surface localization of TMEM43, we performed cell surface biotinylation assay. We found that TMEM43 wild-type (WT) protein trafficked to the plasma membrane, whereas TMEM43-p.(Arg372Ter) showed almost complete disappearance of the biotinylated protein along with a significant reduction in total protein (Fig. 4*C*). Analysis of protein stability by the cycloheximide chase assay implies that this reduction might have been due to a decreased protein stability of TMEM43-p.(Arg372Ter) (Fig. 4*D*). Together, these results demonstrate the putative protein topology of TMEM43 and its localization to plasma membrane.

### Generation of p.(Arg372Ter) Knock-In Mice Displaying Morphological Change in GLSs with Progressive Hearing Loss.

**Fig. 5.**
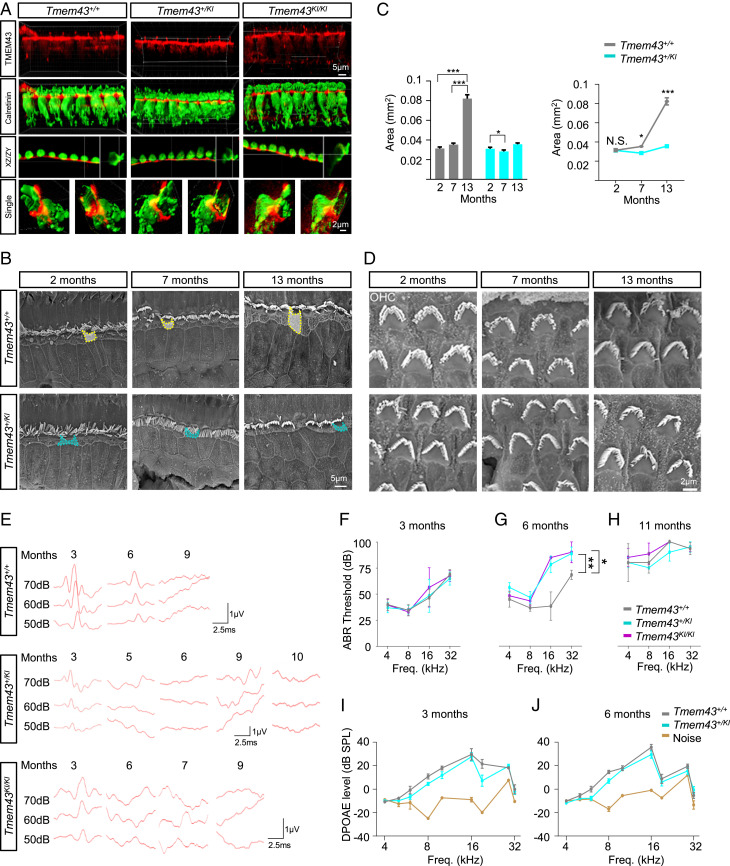
*Tmem43-*p.(Arg372Ter) KI mice display morphological and physiological dysfunction. (*A*) Confocal micrographs of the organs of Corti from 1-mo-old littermates of *Tmem43*^*+/+*^, *Tmem43*^*+/KI*^, and *Tmem43*^*KI/KI*^. Three-dimensional rendered images were obtained from the apical turn of cochlea immunolabeled with anti-TMEM43 (red, first row) and anti-calretinin (green, second row). XZ and ZY section views are shown (third row). Surface object clipping images of a single IHC (fourth row, *Left*) and cut-away object images of the same IHC (fourth row, *Right*) are shown. Cut-away plane is parallel to the cuticular plate. (*B*) sEM findings at the level of reticular lamina of hair cells. Yellow and cyan dotted lines each indicate apical surface area of inner border cells of *Tmem43*^*+/+*^ and *Tmem43*^*+/KI*^, respectively. (*C*) Summary graphs of apical surface area of inner border cells (*n* = 50) from *B*. (*D*) Representative sEM images of whole mounts from apical turns of cochlea showing intact OHC bundles in *Tmem43*^*+/KI*^ mice that are comparable to *Tmem43*^*+/+*^. (*E*) Representative ABR waveforms in response to 16 kHz tone burst sound that are recorded longitudinally at time intervals from the same mice in each genotype. *y*-axis, sound pressure level (SPL). (*F*) Mean ± SEM. ABR thresholds from littermate *Tmem43*^*+/+*^ (*n* = 10), *Tmem43*^*+/KI*^ (*n* = 10), and *Tmem43*^*KI/KI*^ (*n* = 10) at 3 mo. (*G*) Mean ± SEM. ABR thresholds from littermate *Tmem43*^*+/+*^ (*n* = 3), *Tmem43*^*+/KI*^ (*n* = 12), and *Tmem43*^*KI/KI*^ (*n* = 3) measured at 6 mo. (*H*) Mean ± SEM. ABR thresholds from littermate *Tmem43*^*+/+*^ (*n* = 3), *Tmem43*^*+/KI*^ (*n* = 3), and *Tmem43*^*KI/KI*^ (*n* = 3) measured at 11 mo. (*I*) Mean ± SEM growth functions of DPOAE thresholds from 4 to 32 kHz for littermate *Tmem43*^*+/+*^ (*n* = 6) and *Tmem43*^*+/KI*^ (*n* = 6) measured at 3 mo. (*J*) Mean ± SEM growth functions of DPOAE thresholds from 4 to 32 kHz for littermate *Tmem43*^*+/+*^ (*n* = 8) and *Tmem43*^*+/KI*^ (*n* = 8) measured at 6 mo. The DPOAE responses from *Tmem43*^*+/KI*^ are not significantly different from *Tmem43*^*+/+*^ (ANOVA, *P* > 0.05) at whole kHz and significantly higher than noise level (yellow) at the frequencies from 8 to 19 kHz.

To examine the pathogenic effect of mutant TMEM43 on the in vivo function of cochlea, a KI mouse (*Tmem43*^*KI*^) harboring the human variant, p.(Arg372Ter) of *TMEM43* in the corresponding mouse *Tmem43* sequence (C57BL/6J; 129S-*Tmem43*^*tm1Cby*^), was successfully established (*SI Appendix*, Fig. S6 *A* and *B*). The mutant TMEM43 protein was present in the GLSs till the adult period, as shown by a positive immunostaining from *Tmem43*^*KI*^ mouse models (Fig. 5*A* and *SI Appendix*, Fig. S6*C*). Scanning electron microscopic (sEM) examinations showed that the apical surface area of GLSs, specifically the inner border cells of the organ of Corti from *Tmem43*^*+/KI*^, became significantly narrower than that of *Tmem43*^*+/+*^ as early as 7-mo-old (Fig. 5 *B* and *C*). This reduced size of GLSs became more prominent at 13 mo of age in *Tmem43*^*+/KI*^ (Fig. 5 *B* and *C*). In contrast, the shape and the arrangement of hair cell stereocilia as well as the number of synaptic ribbons were not different between the two genotypes (Fig. 5*D* and *SI Appendix*, Fig. S7). The sEM images of OHCs showed no gross differences between stereocilia of *Tmem43*^*+/+*^ and *Tmem43*^*+/KI*^ mice at 2-, 7-, and 13-mo of age (Fig. 5*D*). When number of hair cells were quantified and compared in *Tmem43*^*+/+*^, *Tmem43*^*+/KI*^, and *Tmem43*^*KI/KI*^ mice, neither IHC nor OHC number showed significant difference in 3-mo-old mice (*SI Appendix*, Fig. S8). A slight decrease in the number of IHC (∼15 to 45% segment) was observed only in 7-mo-old *Tmem4*^*KI/KI*^ (*SI Appendix*, Fig. S8). In addition, the spiral ganglion neuron (SGN) density of *Tmem43*^*+/KI*^ was not significantly different from *Tmem43*^*+/+*^ at all cochlear regions examined (*SI Appendix*, Fig. S9). These results together indicate that *Tmem43*^*KI*^ mice display morphological dysfunction mostly in GLSs and possibly IHCs.

To investigate the age-related temporal progression of hearing loss from the *Tmem43*^*KI*^ mice, ABR thresholds at 16 kHz from *Tmem43*^*+/KI*^ and *Tmem43* ^*KI/KI*^ mice were evaluated (Fig. 5*E*). At 3 mo of age, neither significant elevation of ABR thresholds nor reduction of suprathreshold amplitude of ABR wave I from *Tmem43*^*+/KI*^ or *Tmem43* ^*KI/KI*^ mice compared with littermate *Tmem43*^*+/+*^ control mice were observed (Fig. 5 *E* and *F* and *SI Appendix*, Fig. S10*A*). However, we were able to observe a significant distortion of ABR waveforms from *Tmem43*^*+/KI*^ at 5 mo of age at 16 kHz (Fig. 5*E*) and finally noticed a significant elevation of ABR thresholds starting at frequencies of 16 and 32 kHz from both *Tmem43*^*+/KI*^ and *Tmem43*^*KI/KI*^ mice compared with littermate *Tmem43*^*+/+*^ control mice at 6 mo of age (Fig. 5 *E* and *G*). With response to 8 kHz tone burst sounds in which there was no ABR threshold difference among genotypes at 6 mo of age (Fig. 5*G*), significantly reduced suprathreshold amplitude of ABR wave I from *Tmem43*^*+/KI*^ and *Tmem43* ^*KI/KI*^ compared with that from *Tmem43*^*+/+*^ was also noted (*SI Appendix*, Fig. S10*B*). At 9 mo or thereafter, significantly elevated ABR thresholds were observed throughout all frequencies irrespective of genotypes, indicative of presbycusis (Fig. 5 *E* and *H*). These results indicate that the *Tmem4*3^KI^ mice experience progressive hearing loss, just as the ANSD subjects of families HN66 and SB162.

To examine whether the progressive hearing loss of *Tmem4*3^KI^ mice originates from OHC dysfunction, we measured DPOAE responses from 4 to 32 kHz in both *Tmem43*^+/+^ and *Tmem43*^*+/KI*^ mice at 3 and 6 mo. The DPOAE responses from the two genotypes were not significantly different but higher than the noise level at the frequencies from 8 to 19 kHz at both 3 and 6 mo (Fig. 5 *I* and *J*). Given that the ABR threshold of *Tmem43*^*+/KI*^ was significantly higher than that of *Tmem43*^*+/+*^ at 16 kHz (Fig. 5*G*), the DPOAE results indicate a preserved OHC function in these frequency ranges and reflect the characteristics of nonhair cell–related ANSD rather than OHC-related hearing loss, in the *Tmem43*^*KI*^ mice. In other words, the progressive hearing loss in *Tmem43*^KI^ may not originate from OHC. Furthermore, the electrocardiography of the *Tmem43*^+/*KI*^ mice did not show any sign of ARVC (*SI Appendix*, Fig. S11), again confirming a profoundly distinct pathogenic effect of p.(Arg372Ter) compared to p.(Ser358Leu).

### TMEM43-p.(Arg372Ter) Variant Disrupts Prehearing Supporting Cell Conductance, Mediated Primarily by Gap Junctions.

**Fig. 6.**
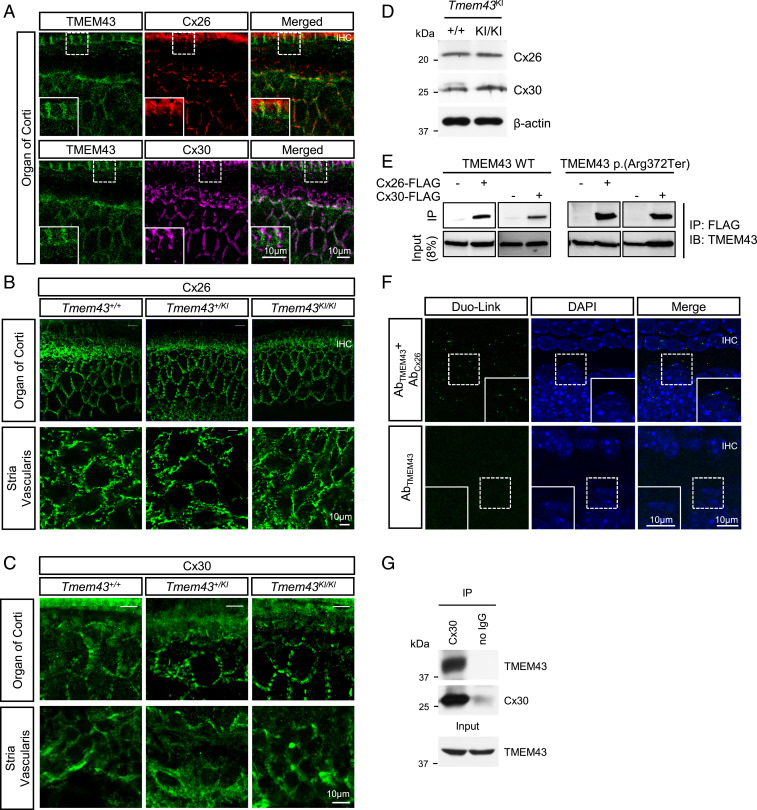
TMEM43 interacts with Cx26 and Cx30 gap junction channels. (*A*) Immunohistochemistry data showing TMEM43 expression with Cx26 and Cx30 in GLSs (P20). Merged images are shown in *Bottom*. TMEM43, green; Cx26, red; Cx30, magenta; DAPI, blue. (*B*) Confocal micrographs of Cx26 expression in the organ of Corti (*Top*) and Stria Vascularis (*Bottom*) of *Tmem43*^*+/+*^, *Tmem43*^*+/KI*^, and *Tmem43*^*KI/KI*^ (5 mo). (*C*) Confocal micrographs of Cx30 expression in the organ of Corti (*Top*) and Stria Vascularis (*Bottom*) of *Tmem43*^*+/+*^, *Tmem43*^*+/KI*^, and *Tmem43*^*KI/KI*^ (7 mo). There was no difference among the genotypes. (*D*) Western blot result from cochlea tissue lysates of *Tmem43*^*+/+*^ and *Tmem43*^*KI/KI*^ mice (p6), blotted with anti-Cx26 and anti-Cx30. (*E*) Co-IP data showing that both TMEM43 WT and TMEM43-p.(Arg372Ter) are immuno-pulled down with Cx26 and Cx30 in in vitro. (*F*) Duolink PLA with anti-TMEM43 and anti-Cx26 (*Top*). Duolink PLA signal was amplified as a red fluorescent, indicative of close proximity of TMEM43 and Cx-26. Red signal was pseudo colored as green for better data display. *Lower* is a negative control data without Cx26 antibody. (*G*) Co-IP data using cochlea tissue (p6). Protein lysate was pulled down with Cx30 antibody and blotted with anti-TMEM43.

TMEM43 has been previously shown to be critical for gap junction channel function in cardiac muscles (35), raising a possibility that pathogenic effect of TMEM43-p.(Arg372Ter) involves gap junction channels in cochlea such as Cx26 or Cx30 (40). Therefore, we examined the localization of TMEM43 with connexin channels. Immunostaining data showed that TMEM43 was expressed in GLSs along with Cx26 and Cx30 (Fig. 6*A*). In addition, the cochlea of *Tmem43*^*+/+*^, *Tmem43*^+/*KI*^, and *Tmem43*^*KI/KI*^ all showed a similar Cx26 and Cx30 expression level (Fig. 6 *B* and *C* and *SI Appendix*, Fig. S12). Cx26 and Cx30 protein expression level in *Tmem43*^*KI/KI*^ was confirmed again with Western blot, which showed similar protein expression level when compared to *Tmem43*^*+/+*^ (Fig. 6*D*). In order to check the protein interaction of TMEM43 with these connexin channels, we next performed coimmunoprecipitation (co-IP) assay in heterologous expression system. Both TMEM43 WT and TMEM43-p.(Arg372Ter) protein were immunoprecipitated with either Cx26 or Cx30 protein when coexpressed in HEK293T cells (Fig. 6*E*). TMEM43 and connexin interaction was further examined in cochlea tissue by Duolink proximity ligation assay (PLA). A positive PLA signal was observed in cochlear GLSs with TMEM43 and Cx26 antibodies but not with TMEM43 antibody alone (Fig. 6*F*), indicating a close proximity of the two proteins (<40 nm). Consistent with the co-IP results in in vitro system, TMEM43 was pulled down with anti-Cx30 antibody in the endogenous cochlea tissue (Fig. 6*G*). These results together indicate that TMEM43 interacts with the connexin channels.

**Fig. 7.**
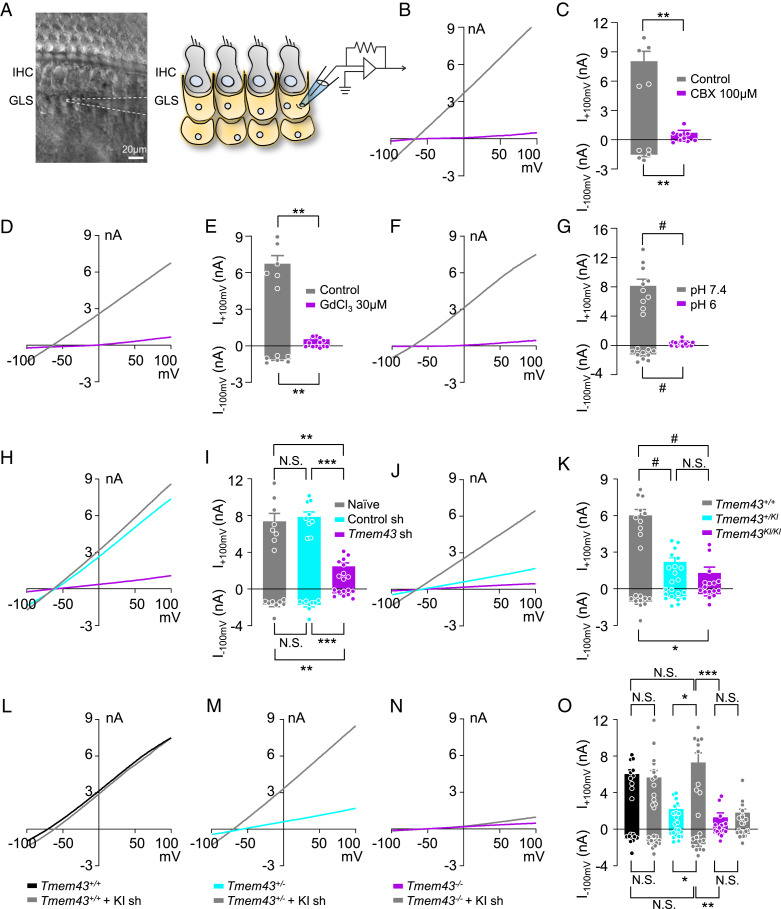
TMEM43-p.(Arg372Ter) variant disrupts prehearing supporting cell conductance, mediated primarily by gap junctions. (*A*) Differential interference contrast (DIC) image of freshly isolated mouse cochlea with glass pipette (white dotted line) whole-cell patch-clamped to a GLS (*Left*). Illustration depicting cochlea tissue with patch-clamped GLS (*Right*). (*B*, *D*, and *F*) Representative current–voltage (I-V) curve measured from GLSs of control (gray) and CBX-treated (*B*), GdCl_3_-treated (*D*) and pH6-treated (*F*) (purple) cochlea. (*C*, *E*, and *G*) Summary bar graph of *B* (*C*), *D* (*E*), and *F* (*G*). (*H*) Representative I-V curve measured from naïve (gray), control shRNA-treated (cyan), and *Tmem43* shRNA-treated (purple) cochlea. (*I*) Summary bar graph of (*H*). (*J*) Representative current–voltage relationship of *Tmem43*^*+/+*^ (gray), *Tmem43*^*+/KI*^ (cyan) and *Tmem43*^*KI/KI*^ (purple). (*K*) Summary bar graph of GLS currents from (*J*). (*L*–*N*) GLS current measured from *Tmem43* KI shRNA uninfected and infected cochlea of *Tmem43*^*+/+*^ (*L*), *Tmem43*^*+/KI*^ (*M*), and *Tmem43*^*KI/KI*^ (*N*) mice. Note that the *Tmem43* KI shRNA rescues impaired passive current of *Tmem43*^*+/KI*^. (O) Summary bar graph of *L*–*N*.

It has been reported that K^+^ channel expression is low in GLSs, so most of their resting membrane conductance is mediated by gap junctions, and gap junction blockers or isolation of GLSs increase their membrane resistance dramatically (10), implying that gap junction channels mediate passive conductance current in GLSs. To test a possible involvement of TMEM43 in connexin channel function, we performed whole-cell patch clamp in GLSs from acutely dissected cochlea tissue (Fig. 7*A*). Indeed, the large passive current with a linear current–voltage relationship was recorded from GLSs. The large passive current was abolished when gap junction channel blocker carbenoxolone (CBX) was treated (Fig. 7 *B* and *C*). As the GLS gap junction networks are known to take part in recirculation of cochlear K^+^ ions (8), we treated a broad-spectrum nonselective cation channel blocker GdCl_3_ (41). Treatment of GdCl_3_ (30 μM) also abolished the passive current of GLSs (Fig. 7 *D* and *E*). We next examined whether pH change can gate the passive conductance of GLS as some connexin channels are reported to be pH sensitive (8). When extracellular pH was lowered to 6, the passive conductance current was completely abolished (Fig. 7 *F* and *G*). These results indicate that GLSs display the large passive conductance current which is cationic and pH sensitive. The similar elimination of the passive conductance current was observed when cultured cochlea tissue was infected with a virus carrying *Tmem43* shRNA (Fig. 7 *H* and *I*). We subsequently measured the passive conductance current from *Tmem43*^*KI*^ mouse. The passive conductance current from GLSs of *Tmem43*^*+/+*^ was significantly reduced by 89% in *Tmem43*^*KI/KI*^ mouse (Fig. 7 *J* and *K*), indicating that TMEM43 contributes majorly to the passive conductance of the GLSs. The heterozygote mouse, *Tmem43*^+/*KI*^, which mimics the counterpart human ANSD subjects, showed a significant reduction of 63% in the passive conductance current in GLSs (Fig. 7 *J* and *K*), confirming the significant dominant-negative pathogenic effect of the TMEM43 variant on the function of GLSs. To rescue the impaired TMEM43-p.(Arg372Ter)–mediated current, we generated an shRNA specifically targeting the KI sequence of *Tmem43*^*KI*^ mouse. This *Tmem43* KI shRNA targets only KI allele but not WT allele (*SI Appendix*, Fig. S5*C* and Fig. 7 *L* and *O*). When *Tmem43*^*+/KI*^ heterozygote cochlea culture was infected with the virus carrying *Tmem43* KI shRNA, the reduced passive conductance current was rescued to the level comparable to *Tmem43*^*+/+*^, due to an unmasking of the remaining WT allele (Fig. 7 *M* and *O*). However, this *Tmem43* KI shRNA did not rescue the passive current from *Tmem43*^*KI/KI*^ cochlea, because these homozygote mice carried no WT allele to be unmasked (Fig. 7 *N* and *O*). These results clearly explain the autosomal dominant effect of TMEM43-p.(Arg372Ter) and emphasize the role of TMEM43 in connexin-linked function in mediating the passive conductance current in GLSs.

### Cochlear Implant Restores the Impaired Speech Discrimination in the ANSD Subjects.

**Fig. 8.**
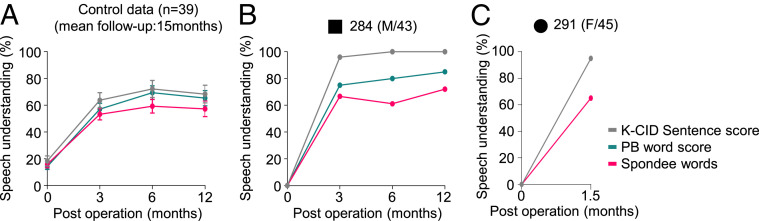
Timely cochlea implantations in family SB162 rescue impaired speech discrimination. (*A*) Longitudinal change of postoperative auditory performance from control subjects (*n* = 39) with adult-onset progressive hearing loss. (*B*) Speech discrimination performance scores of subject #284 measured at 3, 6, and 12 mo following cochlear implantation. (*C*) Speech discrimination performance scores of subject #291 measured at 1.5 mo following cochlear implantation. K-CID Sentence score, Korean Central Institute for the Deaf score; PB word score, Phonetically Balanced word score; Spondee words, a word with two syllables—disyllabic, that is, pronounced with equal emphasis on first and second syllables.

Clinically, determining the exact lesion site of hearing loss is critical for choosing a proper treatment. For example, subjects with conductive hearing loss or moderate sensorineural hearing loss due to limited damages to OHCs would benefit from hearing aids, whereas subjects with severe to profound sensorineural hearing loss, as a result of significant damages to OHCs or IHCs, would require a CI. A subset of sensorineural hearing loss mainly affecting more central structures such as SGN or cochlear nerve or central hearing loss originating from the brainstem or brain may benefit from brainstem implant but not from either hearing aids nor CI. Because the main damage in *Tmem43*^*+/KI*^ mouse was restricted to GLSs, but not to the hair cells or SGNs at minimum 6∼7 mo of age, we could predict that subjects from SB162 could benefit from CI. Consequently, CI was performed on #284 and #291, even though their PTA results did not meet a conventional criterion of CI (PTA equal to or exceeding 70 dB) (42). Postoperatively, the open-set speech understanding of #284 and #291 with a deaf duration of about 15 y restored much more rapidly than that of control adult cochlear implantees (*n* = 39), as measured by speech evaluation test tools (Fig. 8 *A*–*C* and *SI Appendix*, Fig. S13*A*) (43, 44). However, the restoration of speech discrimination ability in #304 with a longer deaf duration (about 25 y) was not as rapid (*SI Appendix*, Fig. S13 *A* and *B*). Notably, the responsiveness of cochlear nerve to stimuli of #304, measured by intracochlear electrically evoked ABR, was restored immediately after cochlear implantation (*SI Appendix*, Fig. S13*C*). These results suggest that limited improvement of speech discrimination in #304 is presumably due to cortical damage related with longer deaf duration but not due to CI-related issues. Based on our results, early cochlear implantation may be recommended for restoration of speech discrimination in the case of TMEM43-p.(Arg372Ter) among late-onset, progressive ANSD subjects.

## Discussion

In this study, we have identified a deafness locus, Chr 3: 13,165,401 to 14,502,000 (3p25.1), and a deafness gene, *TMEM43*, whose variation can cause ANSD post lingually. The unique ANSD phenotype shared by two unrelated families significantly strengthens the causal link between this variant and ANSD. Unbiased exome sequencing screening was performed for both HN66 and SB162 in a parallel fashion with linkage analysis to minimize errors arising from spurious linkages. Significant overlap of linkage interval between HN66 and SB162 in Chr 3: 13,165,401 to 14,502,000 also made it unlikely for our linkage data to be reflection of spurious linkage. Further, we have identified and characterized the role of TMEM43 in connexin-linked function contributing to the passive conductance current in GLSs which is necessary for hearing and speech discrimination (*SI Appendix*, Fig. S14). Until now, only the pathological functions of TMEM43 have been implicated in arrhythmia (34, 35) and tumor progression (45). The variant p.(Ser358Leu) of *TMEM43* has been reported to cause ARVC by decreasing the expression and localization of tight junctions, redistributing the gap junction proteins from the surface to the cytoplasm and decreasing the conduction velocity (35). On the other hand, *Tmem43*^+/KI^ mice did not show any sign of ARVC (*SI Appendix*, Fig. S11), implying that the pathogenic mechanism of ARVC and ANSD would be different. However, we cannot rule out the possibility that underlying mechanism of ARVC and ANSD is independent. This is because even *Tmem43*-p.(Ser358-Leu) variant alone did not show any sign of ARVC in *Tmem43*^*+/Ser358Leu*^ mice, and *Tmem43*^*Δ/Δ*^ mice also did not show any sign of ARVC (46). Thus, absence of ARVC from the *Tmem43*^*+/KI*^ mice does not necessarily support the hypothesis that the underlying mechanism of ARVC and ANSD would be unrelated.

Previous studies have reported the presence of high conductance, octanol-, and flufenamic acid–sensitive GLS currents (47, 48). In this study, we have established a more detailed characterization of the CBX-, GdCl_3_-, and low pH–sensitive and TMEM43-dependent passive conductance current in the GLSs of intact cochlea. In the cochlea, the endocochlear potential of 80 mV is generated by maintaining high K^+^ concentration in the endolymph, and this potential is critical for activation of hair cells (49). The presence of passive conductance current implicates an important role of GLSs in homeostasis of K^+^ and volume regulation. We have observed a significantly smaller inner border cell size of *Tmem43*^*KI*^ mice compared to control, with loss of passive conductance current. We hypothesize that the smaller inner border cells have smaller capacity for K^+^ uptake. The loss of the passive conductance current indicates impaired recycling of K^+^. Such breakdown of K^+^ homeostasis would make hair cells difficult to depolarize, resulting in a disruption of speech discrimination ability. Consistent with this hypothesis, we observed the increasing apical surface area in inner border cell at the age between 7 mo and 13 mo. As one of the major functions of cochlear supporting cells, including inner border cells, is to regulate cochlear K^+^ homeostasis via leak K^+^ channels and gap junction channels, we hypothesized that the increase in inner border cell is to maintain its homeostasis during aging process. The detailed mechanisms await future investigation.

Our study demonstrates that hearing impairment of *Tmem43*^*+/KI*^ and *Tmem43*^*KI/KI*^ begins at some time between 3 and 6 mo (equivalent to twenties in humans). This raises a fundamental question of why canonical ANSD does not occur in younger age. The passive conductance current recordings from the cochlea of P5 to 7 pups already revealed a 63% reduction in passive conductance current in heterozygous *Tmem43*^*KI*^ mice. We also showed that lowering the pH to 6 reduced the passive current of GLS. Thus, the late-onset hearing loss could be explained by reduction of cochlear pH in aging mouse and human, similar to what happens in aging brain (50). Therefore, the normal hearing in heterozygote *Tmem43*^*KI*^ mice and the ANSD subjects at younger age could be due to the remaining passive conductance current that might be enough to maintain homeostatic functions of GLSs. In contrast, the hearing loss in the aging *Tmem43*^*KI*^ mice and ANSD subjects can be explained with further reduction of the remaining current during aging, in which gap junction channels are disturbed. Unfortunately, due to the technical limitation of recording currents from adult cochlea tissue, we could only record the passive conductance current from the cochlea of P5 to 7 pups. The possibility of the remaining passive conductance current in aged WT and *Tmem43*^*KI*^ mice needs to be tested in the future.

Our study provides a potential prognostic genetic marker *TMEM43*-p.(Arg372Ter) for ANSD patients. More importantly, our study suggests that if a causative gene of ANSD is expressed mainly at GLSs, it could also be used as a favorable prognostic marker for CI. Although CI successfully rehabilitates severe to profound sensorineural hearing loss in many cases, adult ANSD patients usually hesitate to receive the surgery due to uncertainty of surgery outcome regarding speech discrimination, side effects including loss of residual hearing and high cost. Given this, an ANSD-causative variant of GLS-specific gene such as p.(Arg372Ter) of *TMEM43* could serve as a useful indicator of positive CI surgery outcome if the surgery is timely done. #284 and #291 who received CI at the age 43 and 50, respectively, recovered the sentence understanding ability to 95 to 100% much more rapidly than did control adult cochlear implantees (*n* = 39). Thus, we may have to recommend the ANSD patients carrying *TMEM43*-p.(Arg372Ter) to receive a CI surgery as soon as they start to experience significantly diminished speech discrimination even though they retain significant residual sound detection ability. Our study provides an important step toward identifying more *TMEM43*-related ANSD patients, calling for further study with larger number of subjects carrying this allele.

In conclusion, we have characterized the role of TMEM43 in connexin-linked function and have delineated that an alteration of this protein leads to functional and morphological abnormalities in GLSs, resulting in the failure to maintain speech discrimination in aged human. Through these mechanistic insights, we have elucidated a link between abnormality in cochlear GLSs and impaired human speech discrimination. We further provide a model platform in which the personalized timing and mode of auditory rehabilitation can be determined, highlighting the importance of a precision medicine–based approach.

## Materials and Methods

### Family Data.

Clinical examination was performed on 20 subjects from Korean family SB162 and 16 subjects from Chinese family HN66. Informed consent was obtained from all participants. This study was approved by the institutional review board of Seoul National University Bundang Hospital (IRB-B-1007-105-402) and the ethics committee of Xiangya Hospital of Central South University (ID: 201603518).

### Animals and Housing.

*Tmem43*-p.(Arg372Ter) KI (C57BL/6J; 129S-*Tmem43*^*tm1Cby*^) mice, 129Sv/Ev mice, C57BL/6J mice, and crab-eating macaque were used. All animals were kept on a 12 h light–dark cycle in a specific-pathogen–free facility with controlled temperature and humidity and had free access to food and water. All experimental procedures were conducted according to protocols approved by the directives of the Institutional Animal Care and Use Committee of Seoul National University Bundang Hospital and the Institutional Animal Care and Use Committee of the Institute for Basic Science.

### Immunocytofluorescence on Cochlear Tissue.

Mouse inner ears (C57BL/6) at various time points postnatally and the cochlea from three monkeys were fixed in ice cold 4% paraformaldehyde for 1 h. Cochlear turns were excised and incubated in blocking buffer and incubated overnight at 4 °C with primary antibodies diluted in the blocking buffer. After three washes, the cochlear turns were reacted with secondary antibodies diluted in blocking buffer for 1 h at room temperature. Samples were then rinsed once with blocking buffer and twice with phosphate-buffered saline. Using Fluorsave reagent, the tissues were mounted on glass slides and covered with coverslip. High-resolution images were obtained using a confocal laser scanning microscope.

### Electrophysiological Recording in Cochlear Supporting Cells.

Cochlea of P5-P7 *Tmem43*-p.(Arg372Ter) KI mouse were isolated in Hepes buffer containing: 144 NaCl, 5.8 KCl, 1.3 CaCl_2_, 2 MgCl_2_, 10 Hepes, 0.7 NaH_2_PO_4_ and 5.6 D-glucose in millimolar (pH 7.4). All recordings were done with same Hepes buffer. Stria vascularis and tectorial membrane were carefully peeled off, and the remaining cellular organization of the organ of Corti was left intact. Acutely dissected cochlea turn was used within 2 h of dissection. Recording electrodes (7 to 11MΩ) supplemented with 126 K-Gluconate, 5 Hepes, 0.5 MgCl_2_, and 10 BAPTA in millimolar (pH 7.3) were advanced through tissue under positive pressure.

### Data Analysis and Statistical Analysis.

Off-line analysis was carried out using Clampfit version 10.4.1.10 and GraphPad Prism version 7.02 software. Significance levels were given as: N.S., *P* > 0.05, *P < 0.05, **P < 0.01, ***P < 0.001, and ^#^*P* < 0.0001.

For full, detailed materials and methods, refer to *SI Appendix*, *Materials and Methods*.

## Supplementary Material

Supplementary File

Supplementary File

Supplementary File

## Data Availability

All study data related to this work are available in the main text and supplementary materials.
