# Supplementary material for: A nonsense TMEM43 variant leads to disruption of connexin-linked function and autosomal dominant auditory neuropathy spectrum disorder
Source: Proc Natl Acad Sci U S A. 2021 May 28;118(22):e2019681118. doi: 10.1073/pnas.2019681118 (PMC8179140; doi:10.1073/pnas.2019681118)
Supplement: Supplementary File [file pnas.2019681118.sapp.pdf]

PNAS
www.pnas.org

Supporting Information for

A nonsense *TMEM43* variant leads to disruption of connexin-linked function and autosomal dominant auditory neuropathy spectrum disorder

Minwoo Wendy Jang[a,b,1], Doo-Yi Oh[c,1], Eunyoung Yi[d,1], Xuezhong Liu[e,f,1], Jie Ling[g,h,1], Nayoung Kim[i], Kushal Sharma[d], Tai Young Kim[b], Seungmin Lee[c], Ah-Reum Kim[c], Min Young Kim[c], Min-A Kim[j,k], Mingyu Lee[l], Jin-Hee Han[c], Jae Joon Han[m], Hye-Rim Park[c], Bong Jik Kim[c], Sang-Yeon Lee[m], Dong Ho Woo[n], Jayoung Oh[c], Soo-Jin Oh[o], Tingting Du[p], Ja-Won Koo[c], Seung-Ha Oh[m], Hyun-Woo Shin[m], Moon-Woo Seong[q], Kyu-Yup Lee[r], Un-Kyung Kim[j,k], Jung Bum Shin[p], Shushan Sang[g], Xinzhang Cai[g], Lingyun Mei[g], Chufeng He[g], Susan H. Blanton[e,f], Zheng-Yi Chen[s], Hongsheng Chen[g], Xianlin Liu[g], Aida Nourbakhsh[e], Zaohua Huang[e], Kwon-Woo Kang[d], Woong-Yang Park[i], Yong Feng[g,t,2], C. Justin Lee[a,b,2], and Byung Yoon Choi[c,2]

[1]These authors contributed equally to this work.

[2]Correspondence and requests for materials should be addressed to B.Y.C. (email: choiby2010@gmail.com) or to C.J.L. (email: cjl@ibs.re.kr) or to Y.F. (fengyong_hn@hotmail.com)

This PDF file includes:

SI Materials and Methods
Figures S1 to S14
Tables S1 to S3
Legends for Movies S1 to S2
SI References

Other supplementary materials for this manuscript include the following:

Movies S1 to S2

Materials and Methods

Family data

Clinical examination was performed on a total of 20 subjects: 8 male and 12 female subjects whose age ranged between 7 and 84 years from family SB162. Five affected subjects with progressive "ANSD" and 11 unaffected subjects in a 5 generation Chinese Han HN66 family also participated in the present study. Informed consent was obtained from all participants in this study. Pure-tone audiometry was carried out to evaluate the air and bone conduction thresholds. The binaural mean pure-tone average of thresholds for air conduction (in dB SPL) was calculated for audiometric frequencies of 0.25, 1, 2, and 4 kHz (PTA 0.25, 1, 2, 4kHz). The speech discrimination score was measured whenever possible. Hearing impairment was additionally assessed by the means of ABRs and DPOAEs. The ABR threshold is a minimal amplitude of sound that can elicit the wave V response. Otoscopic examination and tympanometry with acoustic reflex testing were performed systematically to rule out any conductive hearing impairment. To evaluate the function of outer hair cells, sound pressure of DPOAE and surrounding noise were evaluated using a SmartEP (IHS, Miami, FL). An Etymotic 10B+ microphone was inserted to measure sound pressure in the external ear canal, and pure tone from 3.6 to 14.4kHz was presented using Etymotic ER2 stimulator. Frequencies were acquired with an F2-F1 ratio of 1.22. Stimulation levels ranged from 65 to 25 dB SPL at 10-dB interval. This study was approved by the institutional review board of Seoul National University Bundang Hospital (IRB-B-1007-105-402) and the ethics committee of Xiangya Hospital of Central South University (ID: 201603518).

Linkage analysis

The Infinium Global Screening Array panel (Illumina, San Diego, CA), consisting of 660 thousand of SNPs, was used to conduct a whole genome linkage scan with 18 subjects (10 affected and 8 unaffected) in SB182 and 9 subjects (5 affected and 4 unaffected) in HN66. The genomic DNAs were extracted from the peripheral blood. Multipoint $\log_{10} Odds$ (LOD) scores were computed using MERLIN (version 1.1.2) and was given for all genotyped positions. Analyses were performed assuming the dominant traits based on family history. The disease allele frequency was set to 0.0001, and the penetrance for homozygous normal, heterozygous, and homozygous affected were set to 0.0001, 0.01, and 1.0 respectively. Family members with unclear status were coded as unknown. Because Merlin allows a

maximum of 24 bits for a family by default, the family was split into sub-families having common ancestors in SB162 (**Fig. S1*B***).

Targeted exome sequencing and exome sequencing

Targeted exome sequencing of the critical region 3p25.1 from the linkage analysis for four subjects (SB162-#284, #289, #290, #291) was performed and the candidate variants were listed (**Table S2**). The variants <1% minor allele frequency in ESP6500 and 1000G were initially identified. Only inheritance pattern-matched variants remained, and the variants in dbSNP, but not in flagged dbSNP were filtered out. We used our in-house database having KRGDB_1722 individuals to remove Korean-specific common variants. In addition to single nucleotide variation (SNV), possible presence of copy number variations (CNV) was also identified using Excavator (1). A genome level $\log_2$ ratio for chromosome 3 was investigated. The subject proband was screened for hotspot variants causing hearing loss in Korean and Chinese population via hot spot screening, which include *GJB2*, *SLC26A4* and mitochondrial 12S rRNA. In parallel, blind to the linkage analysis results, exome sequencing on the four subjects (SB162-#284, #289, #290, #291) in SB162 and 2 affected subjects (#36 and #28) and 1 unaffected subject (#22) were performed. Bioinformatics analyses were performed as previously described (1). Thereafter, the final candidate variants in these families were verified using Sanger sequencing. The variants in non-coding regions as well as synonymous variants in coding regions were filtered out. Variants with a minor allele frequency of less than 1% were selected based on the Exome Sequencing Project 6500 (ESP6500), 1000 Genome Project (1000G), Exome Aggregation Consortium (ExAC), and our in-house database containing the exomes of 81 Korean individuals. Based on the autosomal dominant inheritance pattern, single heterozygous variants with sufficient read depths (>10X) and genotype quality (>20) were selected when they were commonly found in all the affected siblings. Further, variants of dbSNP ID with no clinical significance were filtered out. Finally, 20 possible candidate variants in SB162 and 5 candidates in HN66 that co-segregated with ANSD phenotype remained (**Table S1** and **Table S2**). To ascertain the candidate pathogenic variants, Exome Sequencing was performed on two affected subjects (#36 and #28) and one unaffected subject (#22) and the resulting sequences were processed using the pipeline that we have previously described (2). After initial quality control, variants were firstly filtered using the deafness-associated gene list (DAGL) to search for variants in known deafness genes. Then, the remaining variants were filtered according to the criteria as

follows: (1) locating at the coding exons and intron-exon adjacent regions; (2) MAF < 0.1% in multiple databases including the ExAC, the 1000 Genome Project, gnomAD and the ATCG (Annoroad Typical Chinese Genomes) database; (3) heterozygous variants in the form of non-synonymous, non-sense, splice sites, Indels, codon variants; (4) predicted pathogenicity for missense variants using SIFT, MutationTaster, and Polyphen2. Candidate pathogenic variants were further conformed by mapping the filtered variants obtained from Exome Sequencing to the linkage regions.

Whole Genome Sequencing

Whole genome sequencing was performed on 8 DNA samples (SB162-284, 289, 290, 304, 307, 309, 324, 332) using HiSeq2000. Bioinformatics analysis (alignment to the hg19 reference genome, deduplication, local de novo assembly, and variant calling) was performed using BWA, picard, and GATK). Variants firstly were restricted to those in specified regions (chr3:11,883,000-chr3:14,502,000, based on the result of linkage analysis). We then filtered out the variants that were not matched to inheritance patterns, and that were in Korean reference database (KRGDB, KOVA and in-house Samsung Genome Institute control subject database) and the 1000G project.

Evaluation of speech discrimination

Speech performance was evaluated preoperatively, and 3, 6, and 12 months after CI using Korean version of the Central Institute for the Deaf (K-CID) and the Korean PB word by experienced speech-therapist. The Korean PB word included 40 monosyllabic words that are phonetically balanced, and K-CID was composed of sentences to evaluate speech discrimination. K-CID and PB word were presented in sound-field with condition of audio-only and no visual cues. Patients were instructed to repeat the speech stimulus verbally, and the score was measured with the correction ratio (%).

Animals and housing

Tmem43-p.(Arg372Ter) Knock-in (C57BL/6J;129S-*Tmem43*^{tm1Cby}) mice, 129Sv/Ev mice, C57BL/6J mice and crab-eating macaque were used. All animals were kept on a 12 hours light-dark cycle in a specific-pathogen-free facility with controlled temperature and humidity and had free access to food and water. All experimental procedures were conducted according to protocols approved by the directives of the Institutional Animal Care and Use

Committee of Seoul National University Bundang Hospital (Seongnam, Republic of Korea) and the Institutional Animal Care and Use Committee of IBS (Daejeon, Republic of Korea).

Construction of *Tmem43*-p.(Arg372Ter) knock-in mice model

The *Tmem43*-p.(Arg372Ter) knock-in (C57BL/6J;129S- *Tmem43*^{tm1Cby}) mouse model was generated using Clustered Regularly Interspaced Short Palindromic Repeats (CRISPR) technology. In such a model, Arginine (R) was replaced with the STOP codon at position 372 of the TMEM43 protein (gene ID: NM_028766). The criteria used for gRNA selection are the distance to the modification site and the off-target profile. Based on these guidelines, two gRNA candidates (*Tmem43* gRNA1:5'- TTAGAGCAGCCCACAGCGGTCGG-3'; *Tmem43* gRNA2: 5'- CCGATTAGAGCAGCCCACAGCGG-3'), located in proximity to the variant site, were selected and evaluated. To determine gRNA activity, a SURVEYOR assay was performed, using SURVEYOR variant detection kit (IDT), according to the manufacturer's instructions. Based on the validated gRNAs, a single stranded oligodeoxynucleotide donor (ssODN) was designed and synthesized. A 160 bp donor contains two homology arms flanking introduced (C → T) variant in exon 12 corresponding to 1114 position in the *Tmem43* gene. Moreover, to protect the repaired genome from being re-targeted by Cas9 complex, additional variants were introduced corresponding to a second stop codon. The 62 C57BL/6J embryos were injected through a cytoplasmic route with a CRISPR cocktail containing ssODN donor, gRNA transcripts *Tmem43* and Cas9 mRNA. Forty-nine out of 62 embryos passed the quality screening and were implanted into two surrogate CD1 mice. From this round of microinjection, all females became pregnant, and gave birth to nine pups F0. To confirm the germline transmission of the transgene, female founder F0 was mated with the wildtype C57BL/6J male and subjected to F1 breeding to confirm germline transmission of the transgene. All animals were analyzed by PCR and sequencing for the presence of correct point variant at the target site. This *Tmem43*-p.(Arg372Ter) knock-in mice line was maintained in the C57BL/6J;129S mixed genetic background. For genotyping, genomic DNA was extracted from 1- to 2-mm-long tail tips using the DNeasy® Blood & Tissue kit (Qiagen, Hilden, Germany). Genomic DNA (5μl) was analyzed by PCR in a final volume of 50μl in the presence of 25mM $MgCl_2$ and 2mM dNTPs, at 10pM of each primer, and 0.02U of TOYOBO KOD Hot Start DNA polymerase (Invitrogen/Life Technologies, Billerica, Massachusetts, USA) with primers: Forward (5'-CCACAGTGGACTGGTTTCCT-3') and Reverse (5'-GGCTTCACTCCAGCTTTTTG-3') detecting the presence of the knock-in allele

(213 base pairs). After a denaturing step at 95°C for 2 minutes, 35 cycles of PCR were performed, each consisting of a denaturing step at 95°C for 20 seconds, followed by an annealing phase at 59°C for 10 seconds and an elongation step at 72°C for 10 seconds. PCR was finished by a 10-minute extension step at 72°C. Amplified products were confirmed by Sanger sequencing (Macrogen Inc., Seoul, KOR). As the sequence difference between *Tmem43*$^{+/+}$ and *Tmem43*$^{KI/KI}$ was too small to be detected by conventional PCR method, mouse cDNA was amplified with primers (F: 5-gtttatgggcctcaacctcatg-3, R: 5-caggcttcactccagctttttgg-3) and then enzyme digested with BsiEI (Thermo Fisher #FD0894). Genotyping result using this method is shown in **Fig. S6*B***. The expected band size for WT is 90bp and123bp, and for KI/KI is 213bp.

Plasmids

TMEM43 (Myc-DDK-tagged)-Human transmembrane protein 43 (TMEM43) (GenBank accession no. NM_024334.2) was purchased from OriGene (RC200998) and cloned into CMV-MCS-IRES2-EGFP vector using BglII/XmaI sites. h*TMEM43*-p.(Arg372Ter) variant was obtained by performing oligonucleotide-directed mutagenesis using the EZchange site-directed mutagenesis kit (EZ004S, Enzynomics). mTMEM43 (GenBank accession no. NM_028766.2) was obtained by using a RT–PCR based gateway cloning method (Invitrogen) and cloned into CMV-EGFP-C1 vector using BglII/SalI sites. GJB2 (NM_004004) Human Tagged ORF Clone was purchased from OriGene (RC202092) and Cx30-msfGFP was purchased from Addgene (69019).

Heterologous expression of TMEM43 on HEK293T cell lines

Human embryonic kidney (HEK) 293T cells were purchased from ATCC (CRL-3216). The cell line has been tested for mycoplasma contamination. HEK293T cell was cultured in DMEM (10-013, Corning) supplemented with 10% heat-inactivated fetal bovine serum (10082-147, Gibco) and 10,000 units/ml penicillin–streptomycin (15140-122, Gibco) at 37˚C in a humidified atmosphere of 95% air and 5% CO_2. Transfection of expression vectors was performed with Effectene Transfection Reagent (Effectene, 301425, Qiagen), according to the manufacturer's protocol. One day prior to performing the experiments, HEK293T cells were transfected with each DNA 1.5µg per 60mm dish. Ratio of DNA to Effectene Reagent is 1:10.

RT-PCR

The 129Sv/Ev mice were deeply anesthetized with pentobarbital sodium (50mg/kg). Total RNA was extracted from their whole cochlea (P1), heart (P120), eye (P42), brain (P28) and kidney (P28) to analyze the expression of the target mRNAs using TRIZOL® (Gibco BRL, Gaithersburg, MD, USA) and a column from a RNeasy Mini Kit (Qiagen, Valencia, USA), performed in accordance with the manufacturer's instructions. The RNA obtained was used to synthesize cDNA with oligo (dT) primers, one-twentieth of which was used for one PCR reaction. The DNA amplification was performed in a final volume of 30μl. PCR cycling conditions were 95°C for 2 min, followed by 35 cycles of 95°C for 20 s, 55°C for 10 s, and 70°C for 10 s, with a final step of 72°C for 5 min. Amplified product (15μl) was separated using electrophoresis in a 2% agarose gel and visualized with ethidium bromide. We analyzed the transcripts of TMEM43 and GAPDH by primer pairs, as follows: the forward primer (5′-CTTCCTGGAACGGCTGAG-3′) and the reverse primer (5′-CACCAGCCTTCCTTCATTCT-3′) for *Tmem43*, and the forward primer (5′-ACCACAGTCCATGCCATCAC-3′) and the reverse primer (5′-CACCACCCTGTTGCTGTAGCC-3′) for *Gapdh*.

Immunocytofluorescence on cochlear tissue

Primary antibodies used are rabbit anti-TMEM43 polyclonal (1:100, NBP1-84132, Novus), mouse anti-mCherry monoclonal (1:500, ab125096, Abcam), mouse anti-calretinin monoclonal (1:500, MAB1568, Millipore), goat anti-Na^+, K^+-ATPase-α3 polyclonal (NKA, 1:250, SC-16052, Santa Cruz), and mouse anti-CtBP2 monoclonal (1:500, 612044, BD Transduction Laboratories), rabbit anti-CtBP2 polyclonal (1:500, BS2287, Bioworld Technology), mouse anti-GluA2 monoclonal (1:500, MAB397, Millipore), mouse anti-connexin 26 monoclonal (1:250, 13-8100, Thermo Fisher), goat anti-connexin 30 polyclonal (1:250, NBP-45247, Novus), chicken anti-GFAP polyclonal (1:250, AB5541, Millipore), and rabbit anti-myosin 6 polyclonal (1:500, M5187, Sigma-Aldrich) antibodies. Secondary antibodies generated either in donkeys or goats were used at 1:1000. Secondary antibodies used are donkey anti-mouse Alexa Fluor 555 (A-31570, Thermo Fischer Scientific), donkey anti-rabbit Alexa Fluor 488 (A-21206, Thermo Fischer Scientific), donkey anti-goat Alexa Fluor 633 (A-21082, Thermo Fischer Scientific), donkey anti-chicken Alexa Fluor 488 (703-545-155, Jackson IR), goat anti-rabbit Alexa Fluor 555 (A-21428, Thermo Fischer Scientific), goat anti-mouse Alexa Fluor 488 (A-11001, Thermo Fischer Scientific), goat anti-

rabbit IgG (H+L) Alexa Fluor 488 (A11008, Life technologies). No immunoreactivity was found when the primary antibodies were omitted. Mouse inner ears (C57BL/6) at various time points postnatally and the cochlea from three monkeys weighing 5kg were fixed in ice cold 4% paraformaldehyde, for 1h. Cochlear turns were carefully excised and incubated in blocking/permeabilizing buffer (PBS with 5% goat or donkey serum and 0.25% Triton X-100). Then, the preparations were incubated overnight at 4°C with primary antibodies diluted in the blocking/permeabilizing buffer. After 3 washes, the cochlear turns were reacted with fluorescence labeled secondary antibodies diluted in blocking/permeabilizing buffer for 1h at room temperature. Samples were then rinsed once with blocking/permeabilizing buffer and twice with PBS. Using Fluorsave reagent (Calbiochem, 345789), the tissues were mounted on glass slides and covered with coverslip. Specific immunolabeling was initially examined under epifluorescence microscope and high-resolution images were obtained using a confocal laser scanning microscope (LSM710, Zeiss).

Hair cell counting and cochleogram

A protocol adapted from previously described (3) was employed. To extract the entire cochlear turns, post-fixed cochleae (4% paraformaldehyde at 4°C for 1h) were transferred into 5% EDTA and incubated at 4°C under gentle agitation for 36-48h. The decalcified otic capsule, the lateral wall, Reisner's membrane and tectorial membrane were carefully removed. The remaining organ of Corti preparation was cut into 2-3 pieces and subjected to the immunolabeling procedure described above (*see* Immunocytofluorescence on cochlear tissue). A series of confocal images of labeled hair cells were obtained from each cochlea using laser scanning confocal microscope (LSM710, Zeiss). The entire length of the organ of Corti was reconstructed by overlapping the common cells at the edges of the individual images in ImageJ. The reconstructed image was then divided into segments, each spanning 1% of the total length. The number of IHCs and OHCs in each segment vs the relative distance from the apex were plotted. We found no significant difference in the length of cochlea among different genotypes. The total length of the organ of Corti is 5.6 ± 0.3mm, which is comparable to the reported value for C57BL/6 mouse (4). The segments that had been damaged during tissue preparation were identified under DIC optics and excluded from hair cell counting analysis.

SGN density analysis

For cochlear tissue sectioning, decalcified cochleae were equilibrated in 30% sucrose solution overnight, embedded in OCT compound (Sakura, 4583), and rapidly frozen by immersing into liquid nitrogen. Frozen blocks were sectioned (20µm thickness) using a cryostat (Leica, CM1860) and mounted on slide glass (Fisher scientific, 22-037-246). After brief rinses with PBS, the frozen sections were immunolabeled with anti-calretinin (Chemicon, MAB1568) and anti-neurofilament (Abcam, AB4680) using the procedure described above (*see* immunocytofluorescence on cochlear tissue). For each acquired confocal-z-stack images, the number of neurofilament-positive neurons was counted at the apical, middle, and basal turns of the cochlea. The SGN density was calculated by dividing the number of neurons by the area occupied by the SGN cell bodies.

Immunocytofluorescence on heterologous system

For immunocytochemistry, h*TMEM43*-IRES2-EGFP was transfected into HEK293T cells one day prior to staining. Cells were fixed in 4% paraformaldehyde for 30 min at room temperature and washed 3 times with PBS. The permeabilized group contained 3% Triton X-100 in the blocking solution with 2% goat serum and 2% donkey serum but the impermeabilized group excluded Triton X-100 in the blocking solution. Cells were incubated with rabbit anti-TMEM43 polyclonal antibody (1:100, NBP1-84132, Novus) and mouse anti-FLAG (1:500, F1804, Sigma-Aldrich) for overnight. After washing, goat anti-rabbit IgG Alexa Flour 647 (1:1000, A21429, Invitrogen) and donkey anti-mouse Alexa 555 (1:1000, A31570, Molecular Probe) were added and incubated for 2 hours at room temperature. The cells were washed 3 times and mounted, and then observed under a Nikon A1 confocal microscope.

***In situ* hybridization**

To make specific riboprobe for mRNA of *Tmem43* and *Spmx*, we cloned partial cDNA fragments of *Tmem43* and *Smpx*. Primers were as follows: *Tmem43*, forward: 5′-TGTTCGTGGGGCTAATGACC-3; reverse: 5′-TAGTTGGTCACCTCGTTGCC-3′, T7-reverse primer: 5′-TAATACGACTCACTATAGGGAGATCACAGTAACCACGTGAGCC-3′; *Smpx*, forward: 5 ′-TTTCCACACGGTCAAGCCTTT-3; reverse: 5′-TCTCCTCAAAACCACACTTCCC-3′, T7-reverse primer: 5′-TAATACGACTCACTATAGGGAGAGTCTTGGGCATTCAGGAGGTT-3′. The plasmid was linearized and used for *in vitro* transcription (Roche Diagnostics, Indianapolis, IN, USA)

to label RNA probes with digoxigenin-UTP. The 3' and 5' - DIG labeled miRCURY LNA miRNA Detection probes (Qiagen, negative control Scramble-miR cat. # YD00699004-BCG) were used for *in situ* hybridization. *In situ* hybridization was performed as previously described (5) with some modifications. Frozen cochlea was sectioned at 20µm thickness on a cryostat. The sections were then fixed in 4% paraformaldehyde, washed with PBS, and acetylated for 10 min. The sections were incubated with the hybridization buffer (50% formamide, 4X SSC, 0.1% CHAPS, 5mM EDTA, 0.1% Tween-20, 1.25X Denhartdt's, 125µg/ml yeast tRNA, 50µg/ml Heparin) and digoxigenin-labeled probes (200ng) for 18h at 60°C. Non-specific hybridization was removed by washing in 2X SSC for 10min and in 0.1X SSC at 50°C for 15min. For immunological detection of digoxigenin-labeled hybrids, the sections were incubated with anti-digoxigenin antibody conjugated with alkaline phosphatase (Roche Diagnostics) for 1h, and the color reaction was carried out with NBT/BCIP (4-nitroblue tetrazolium chloride/bromo-4-chloro-3-indolyl phosphate; Sigma-Aldrich). Sections were dehydrated and mounted with Vectamount (Vector Laboratories, Burlingame, CA, USA).

Cochlear organotypic culture and shRNA treatment

Neonatal mouse (C57/BL6, P5) cochlear turns were isolated in ice cold sterile Hanks' Balanced Salt Solution (HBSS). Cochlear segments were attached on Cell-Tak (354240, Corning) coated coverslips and incubated overnight in DMEM/F12 medium containing 1% fetal bovine serum, 5µg/ml ampicillin, B27 and N2 (37°C, 5% CO_2 humidified incubator). Upon confirming stable attachment, the tissues were treated with either scramble or shRNA diluted in culture medium (1:1000) for 48 h. The medium was then replaced with a fresh one. After additional 48h of incubation, the tissues were subjected to further examination.

Construction and packaging of shRNA–lentiviral vectors

For TMEM43 gene silencing, candidate for both mouse and rat *Tmem43* shRNA (GenBank accession no. NM_028766.2 and NM_001007745.1) sequences were cloned into lentiviral pSicoR vector using XhoI/XbaI sites, as previously described (6). Knockdown efficiency was determined by a reduction in fluorescence expression of CMV-EGFP- TMEM43 when overexpressed with *Tmem43* shRNA candidate containing pSicoR-*Tmem43* shRNA-mCh vector. Validated shRNA candidate containing pSicoR vector was packaged into high-titer lentivirus by KIST virus facility (Republic of Korea). In brief, the lentiviral vectors were

produced by co-transfecting each pSicoR vector with the ViraPower lentiviral packaging mix (Invitrogen) in the 293FT packaging cell line. The supernatant was collected and concentrated by ultracentrifugation. For efficient shRNA delivery, lentiviral vectors were always produced at subneutral pH (≤pH 7.0). The target sequence of *Tmem43* shRNA is 5’-GCTCTTGTCTGACCCAAATTA-3’ and *Tmem43* KI shRNA is 5’-CTCTTCTACTGATGACTGTGG-3’. For control shRNA, scrambled sequence 5’-TCGCATAGCGTATGCCGTT-3’ was inserted in place of shRNA sequence.

Chemicals

Chemicals used in this study were all purchased from Sigma-Aldrich.

Electrophysiological recording in cochlear supporting cells

Cochlea of P5-P7 *Tmem43*-p.(Arg372Ter) Knock-in mouse pups were isolated in HEPES buffer containing (mM): 144 NaCl, 5.8 KCl, 1.3 $CaCl_2$, 2 $MgCl_2$, 10 HEPES, 0.7 NaH_2PO_4 and 5.6 D-glucose (pH 7.4 was adjusted with NaOH). All recordings were done with same HEPES buffer as external solution. Stria vascularis and tectorial membrane were carefully peeled off and the remaining cellular organization of the organ of Corti was left intact. Acutely dissected cochlea turn was used within 2 hours of dissection. Glia-like supporting cells that are located below inner hair cell layer were whole cell patch clamped and current traces were elicited by 1 sec ramps ascending from -100mV to +100mV with -60mV holding potential. Recording electrodes (7-11MΩ) supplemented with (mM): 126 KOH, 126 Gluconate, 5 HEPES, 0.5 $MgCl_2$ and 10 BAPTA (pH adjusted to 7.3 with KOH) were advanced through tissue under positive pressure. Slice chamber was mounted on the stage of an upright Hamamatsu digital camera viewed with an 60X water immersion objective with infrared differential interference contrast optics using Imaging Workbench Software. Electrical signals were digitized and sampled with Digidata 1320A and Multiclamp 700B amplifier (Molecular Devices) using pCLAMP 10.2 software. Data were sampled at 10 kHz and filtered at 1 kHz. Glass pipette were pulled from micropipette puller (P-97, Sutter Instrument) and all experiments were conducted at a room temperature of 20-22°C. For experiments with drug (carbenoxolone, $GdCl_3$) and acidic solution treatment, cochlea tissue was replaced every time with fresh tissue. For experiments with shRNA treatment and transgenic mice, average of 3 cells were patched from a cochlea tissue. In the bar graphs,

outward current (measured at +100mV) as a positive current and inward current (measured at -100mV) as a negative current were plotted in the same bar graph.

Cell surface biotinylation, co-immunoprecipitation and western blot in *in vitro*

For biotinylation, CMV-h*TMEM43* WT-IRES2-EGFP or CMV-h*TMEM43*-p.(Arg372Ter)-IRES2-tdTomato vector were transfected into HEK293T cells 1 day prior to the experiment day. Transfected cells were washed three times with PBS and cell surface-expressed proteins were biotinylated in PBS containing Ez-link sulfo-NHS-LC-Biotin (21335, Thermo) for 30 min. After biotinylation, cells were washed with quenching buffer (100mM glycine in PBS) to remove excess biotin and then washed three times with PBS. The cells were then lysed and incubated with high capacity NeutrAvidin-Agarose Resin (29204, Thermo Fisher). After three washes with lysis buffer, bound proteins are eluted by the SDS sample buffer and subjected to western blot analysis. Primary antibody used is: rabbit anti-TMEM43 polyclonal (1:100, NBP1-84132, Novus). Secondary antibody used is: Donkey anti-rabbit HRP (NA9340, Amersham). For CHX assay, 40μg/ml CHX was treated to TMEM43 WT and p.(Arg372Ter) expressing HEK293T cells and analyzed in 3 hours interval. Anti-FLAG M2 (1:2000, F1804, Sigma-Aldrich) was used for western blot. For co-immunoprecipitation, cell lysates were prepared in a buffer containing 50mM Tris-HCl (pH 7.5), 150mM NaCl, 1% NP-40, 10mM NaF, and protease and phosphatase inhibitor cocktail. Equal amounts of precleared cell lysates were incubated with rabbit anti-FLAG (2368s, Cell Signaling) overnight. Protein A/G-Agarose beads (Thermo Fisher) were added to the mixtures and further incubated for 2h, followed by a wash with lysis buffer. Bound proteins were eluted from the beads with SDS-PAGE sample buffer and western blotting was performed with rabbit anti-TMEM43.

Co-immunoprecipitation and western blot in cochlea tissue

Cochlear tissues of average 20 mice (p6) per sample were extracted and homogenized with Pierce™ IP Lysis Buffer (87787, Thermo Fisher) and Halt™ Protease and Phosphatase Inhibitor Cocktail (100X) (78446, Thermo Fisher). Equal amounts of precleared lysates were incubated with mouse anti-Cx26 (33-5800, Thermo Fisher) and rabbit anti-Cx30 (71-2200, Thermo Fisher) overnight. Protein A/G-Agarose beads were added to the mixtures and further incubated for 2h, followed by a wash with lysis buffer. Bound proteins were eluted from the beads with SDS-PAGE sample buffer and western blotting was performed with

rabbit anti-TMEM43 (1:250). Western blot was performed by blotting cochlea lysate with mouse anti-Cx26 (1:125, 33-5800, Thermo Fisher) and rabbit anti-Cx30 (1:125, 71-2200, Thermo Fisher).

Duolink® PLA

Duolink® *In Situ* Red Starter Kit Mouse/Rabbit (DUO92101, Sigma-Aldrich) was used. On the day of the experiment, cochlea tissue (p6) was obtained, fixed with 4% paraformaldehyde, and washed with 0.3% Triton-X containing PBS. After blocking, sample was incubated with rabbit anti-TMEM43 polyclonal (1:100, NBP1-84132, Novus) and mouse anti-Cx26 monoclonal (1:100, 13-7100, Thermo Fisher) at 4 ˚C, overnight. On the next day, sample was incubated with anti-rabbit MINUS and anti-mouse PLUS probe, ligase and polymerase sequentially. DNA strands participate in rolling circle DNA synthesis only when 2 probes are in close proximity (<40nm). Fluorescent-labeled complementary oligonucleotide probes were observed under Zeiss confocal microscopy.

Scanning electron microscope (SEM) analysis

For SEM analysis, the organ of Corti were performed using a method previously described (7). Briefly, the cochleae were immediately isolated from the euthanized $Tmem43^{+/+}$ and $Tmem43^{+/KI}$ mice at 2, 7, and 13 months of age and fixed in a solution of 2% PFA dissolved in 0.1M sodium cacodylate buffer (pH 7.4) containing 2.5% glutaraldehyde for 1 hour at room temperature. The bony capsule was dissected out and the lateral wall, Reissner's membrane, and tectorial membrane were removed. The organ of Corti was then dissected and fixed in a solution of 0.1M sodium cacodylate buffer (pH 7.4), 2mM calcium chloride, 2.5% glutaraldehyde, and 3.5% sucrose for overnight at 4°C. Following fixation, samples were prepared for SEM using the osmium tetroxide-thiocarbohydrazide (OTOTO) method. Then, the specimens were dehydrated in a graded ethanol solution, dried using a critical point dryer (HCP-2, Hitachi, Japan), attached on the stub, and then coated with platinum using a sputter coater (E1030, Hitachi, Japan). The surfaces of the organ of Corti were captured under a cold-field emission SEM (SU8220, Hitachi, Japan) that was operated at 10 or 15kV. Micrograph measurements were performed using ImageJ software (National Institutes of Health, http://rsbweb.nih.gov/ij/), via the polygon tool for cell area measurements.

Bioinformatics

In silico prediction Algorithm: Polyphen-2 (http://genetics.bwh.harvard.edu/pph2/index.shtml); SIFT (http://sift.jcvi.org/www/SIFT_chr_coords_submit.html); Conservation tools: GERP++ score in the UCSC Genome Browser (http://genome-asia.ucsc.edu/); ExAC, Exome Aggregation Consortium (http://exac.broadinstitute.org/);1000 Genomes (https://www.ncbi.nlm.nih.gov/variation/tools/1000genomes/); KRGDB, Korean Reference Genome DB (http://152.99.75.168/KRGDB/); TOPMED, Trans-Omics for Precision Medicine (https://www.nhlbiwgs.org/); gnomAD (http://gnomad.broadinstitute.org/); MutationTaster (http://mutationassessor.org). Secondary structure prediction of TMEM43 was done with 'TMHMM v2.0' server (http://www.cbs.dtu.dk/services/TMHMM-2.0/). TMEM43 multiple amino acid sequence alignment was done by 'Clustal Omega version 1.2.4' server (https://www.ebi.ac.uk/Tools/msa/clustalo/) and phylogenetic analyses were conducted in 'MEGA version 5' (8). The Human Gene Mutation Database (HGMD®, http://www.hgmd.cf.ac.uk/ac/index.php).

Data analysis and statistical analysis

Off-line analysis was carried out using Clampfit version 10.4.1.10 and GraphPad Prism version 7.02 software. When comparing between two samples, significance of data was assessed by Student's two-tailed unpaired t-test when samples showed normal distribution and assessed by Mann-Whitney test when samples did not pass normality test. Samples that passed normality test but not equal variance test were assessed with Welch's correction. Comparing more than 2 samples were analyzed using one way ANOVA with Tukey's post-hoc test when data passed normality test and Kruskal-Wallis test with Dunn's post-hoc test when data did not pass normality test. Significance levels were given as: N.S. $P>0.05$, *$P<0.05$, **$P<0.01$, ***$P<0.001$ and #$P<0.0001$.

Fig. S1.

A

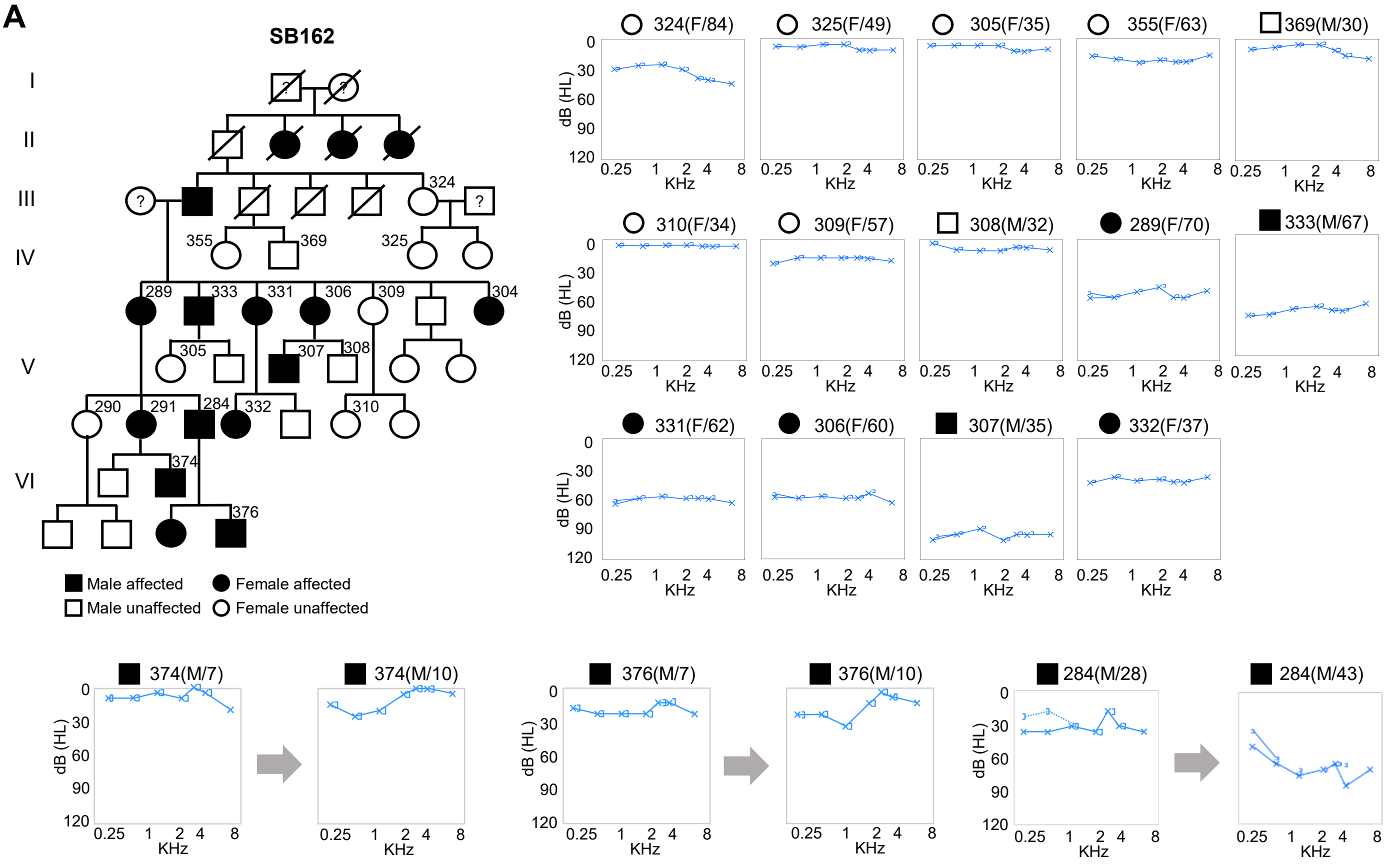
SB162
I
II
III
IV
V
VI
324
355
369
325
289
333
331
306
309
304
305
307
308
290
291
284
332
310
374
376
Male affected
Female affected
Male unaffected
Female unaffected
324(F/84)
325(F/49)
305(F/35)
355(F/63)
369(M/30)
310(F/34)
309(F/57)
308(M/32)
289(F/70)
333(M/67)
331(F/62)
306(F/60)
307(M/35)
332(F/37)
374(M/7)
374(M/10)
376(M/7)
376(M/10)
284(M/28)
284(M/43)
dB (HL)
0
30
60
90
120
0.25
1
2
4
8
KHz

B

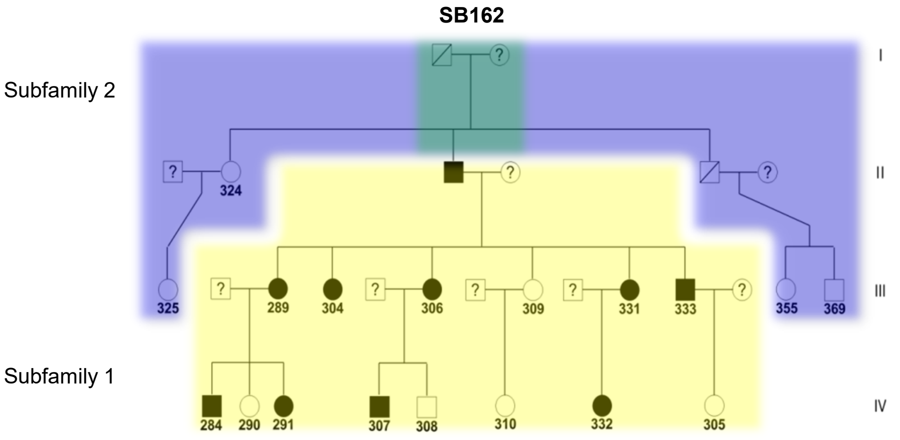
SB162
Subfamily 2
Subfamily 1
I
II
III
IV
324
325
289
304
306
309
331
333
355
369
284
290
291
307
308
310
332
305

C

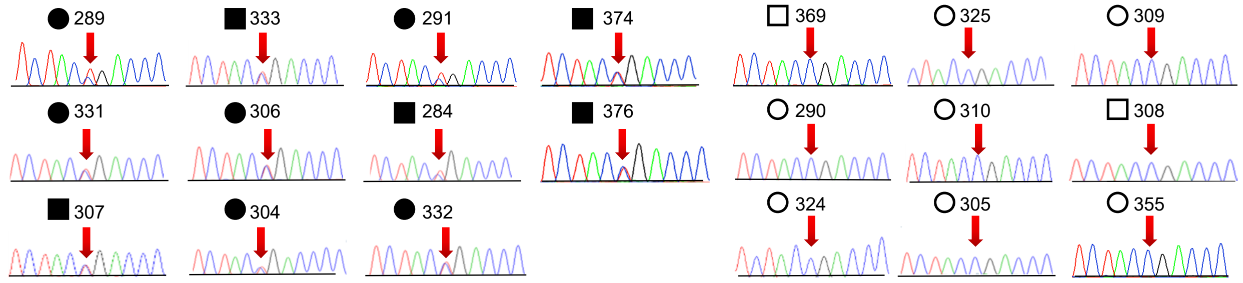
289
333
291
374
369
325
309
331
306
284
376
290
310
308
307
304
332
324
305
355

Figure S1. A p.(Arg372Ter) variant of TMEM43 is the cause of hearing loss in family SB162 as documented by linkage analysis, and segregation study among family members.
(*A*) PTA results from family SB162. Six subjects (#289, #306, #307, #331, #332, #333) show ANSD with 40dB or more degree of PTA as calculated by averages of pure tone thresholds of 500Hz, 1kHz, 2kHz and 4kHz. The auditory thresholds of other subjects stay within a normal range. PTA results in time series from 3 individuals (#284, #374 and #376) show a time-dependent progression of hearing loss. The open and filled symbols indicate unaffected and affected subjects, and square and circular symbols indicate male and female subjects. Subject numbers are superscripted. (*B*) Subfamily 1 and 2 from family SB162 are shaded in yellow and violet, respectively. A common ancestor shared by two subfamilies is shaded in green. (*C*) Representative DNA sequence chromatograms of c.1114C>T variant in TMEM43 from family SB162. Allele c.1114C is indicated in red arrow on the chromatograms.

Fig. S2.

A

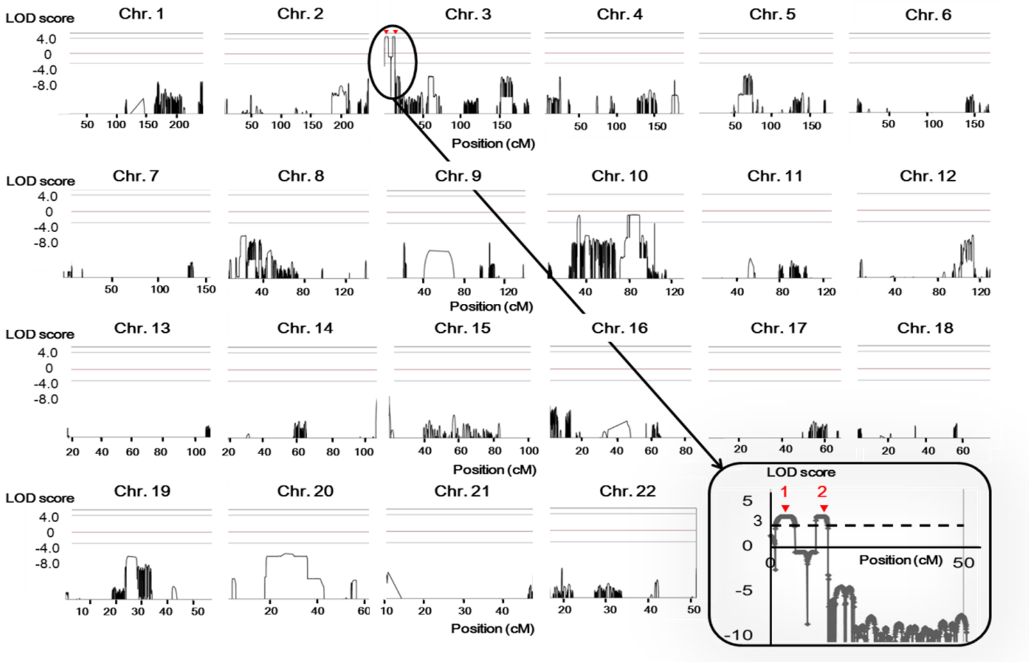
LOD score
Chr. 1
Chr. 2
Chr. 3
Chr. 4
Chr. 5
Chr. 6
Position (cM)
Chr. 7
Chr. 8
Chr. 9
Chr. 10
Chr. 11
Chr. 12
Chr. 13
Chr. 14
Chr. 15
Chr. 16
Chr. 17
Chr. 18
Chr. 19
Chr. 20
Chr. 21
Chr. 22
LOD score
Position (cM)

B

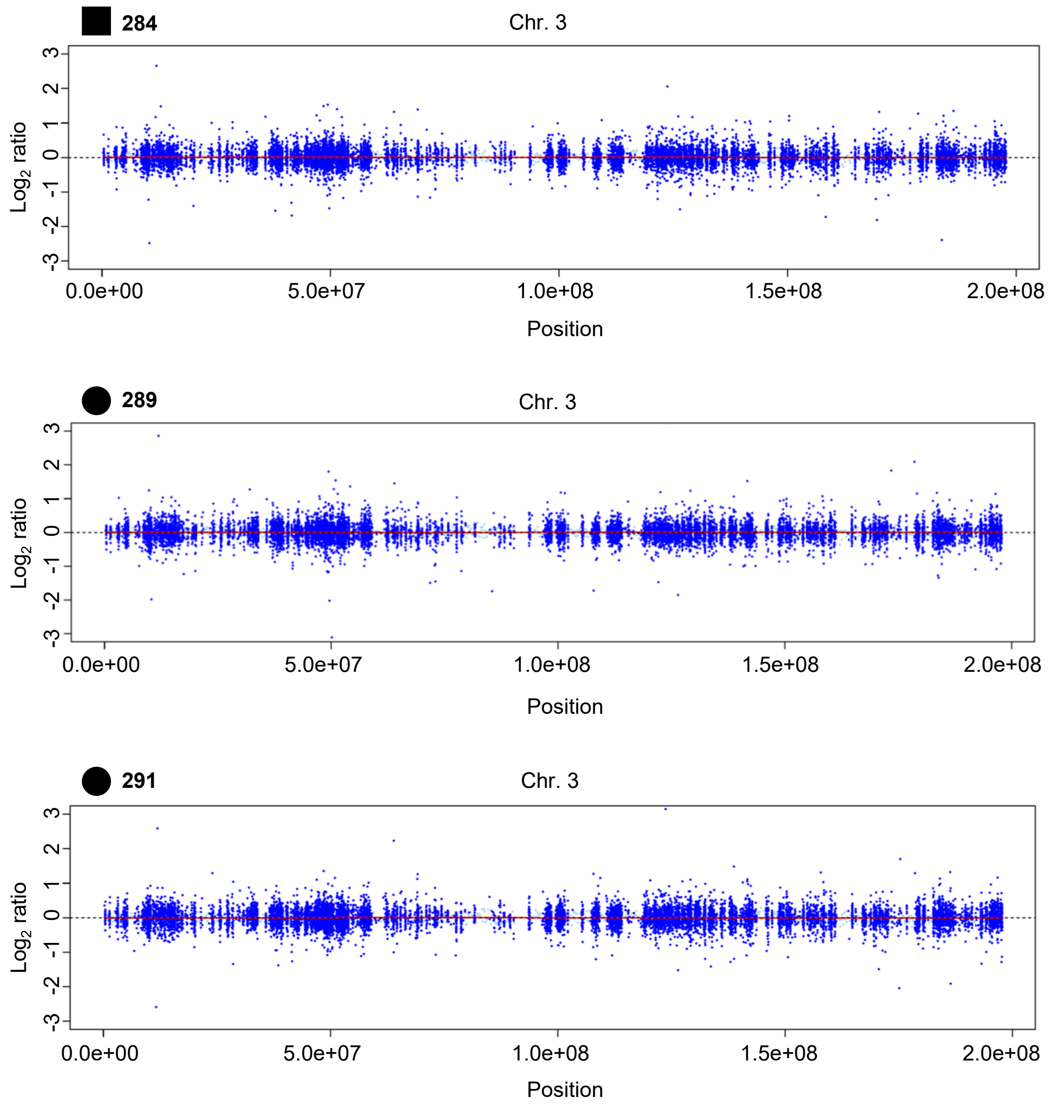
284
Chr. 3
Log2 ratio
Position
289
Chr. 3
Log2 ratio
Position
291
Chr. 3
Log2 ratio
Position

Figure S2. ***TMEM43*-p.(Arg372Ter) is mapped to chromosome 3, without changing copy number variation in family SB162.**

(*A*) Merlin multipoint linkage analysis demonstrates LOD scores greater than 3.0 only on chromosome 3p25-26. Inset: magnified view of two regions on Chr. 3, spanning 6.7cM and 3cM, respectively. Red arrowhead 1: region #1 (Chr. 3: 1,946,000-5,956,000), Red arrowhead 2: region #2 (Chr. 3:11,883,000-14,502,000). (*B*) Presence of copy number variation checked by Excavator. Each panel shows a genome level $\log_2$ ratio for chromosome 3. There is no significant copy number variation.

Fig. S3.

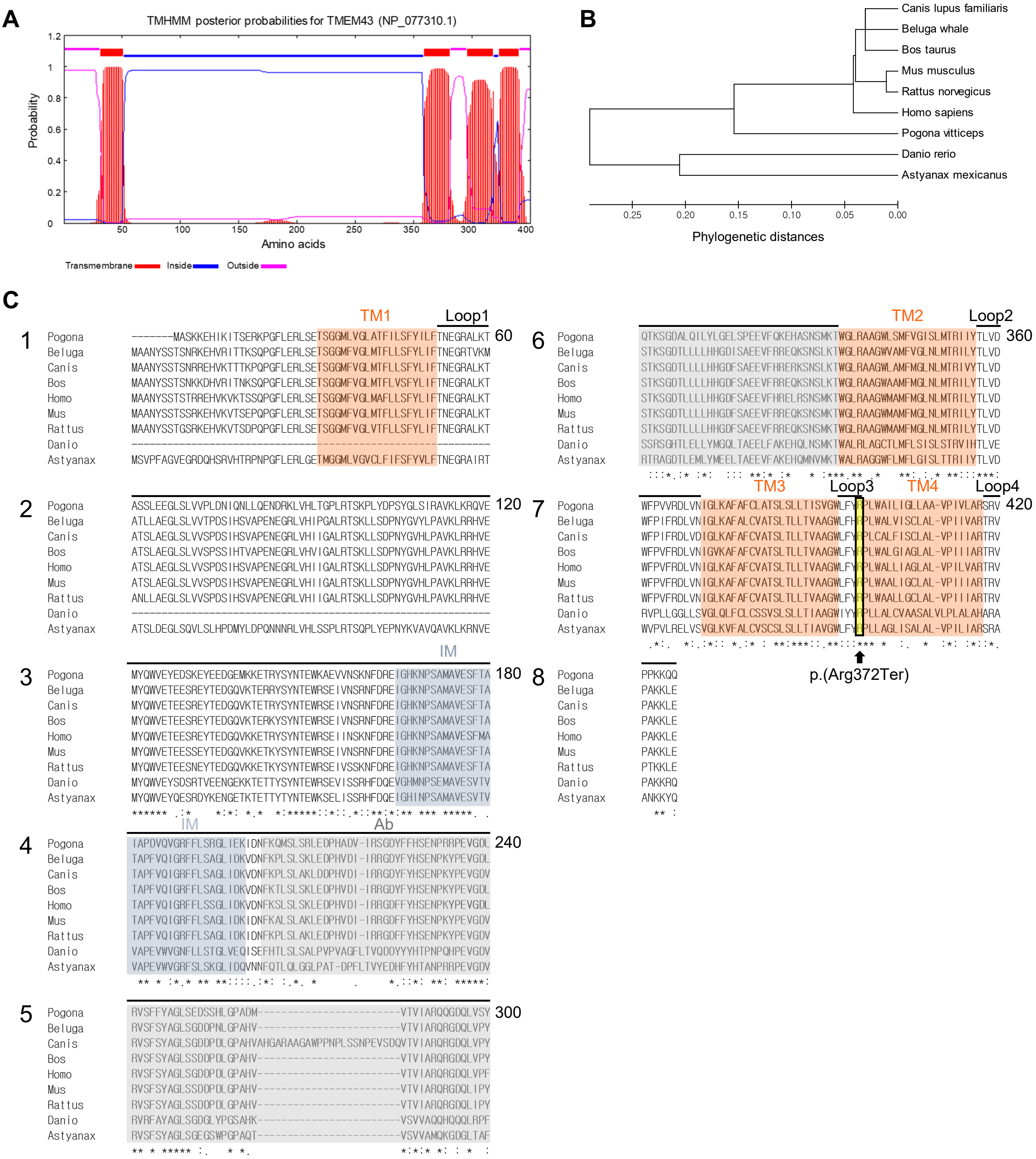
A
TMHMM posterior probabilities for TMEM43 (NP_077310.1)
Probability
Amino acids
Transmembrane
Inside
Outside
B
Canis lupus familiaris
Beluga whale
Bos taurus
Mus musculus
Rattus norvegicus
Homo sapiens
Pogona vitticeps
Danio rerio
Astyanax mexicanus
0.25
0.20
0.15
0.10
0.05
0.00
Phylogenetic distances
C
TM1
Loop1
TM2
Loop2
TM3
Loop3
TM4
Loop4
IM
Ab
Pogona
Beluga
Canis
Bos
Homo
Mus
Rattus
Danio
Astyanax
p.(Arg372Ter)

Figure S3. Phylogenetic analysis of TMEM43 amino acid sequences and structure prediction based on Hydrophobicity analysis.

(*A*), Secondary structure prediction of *homo sapiens* TMEM43 using 'TMHMM v2.0' server. (*B*) The phylogenetic relationship among the 9 different species, inferred using the Neighbor-Joining method by MEGA version 5 software. The optimal tree with the sum of branch length = 1.07711891 is shown. The tree is drawn to scale, with branch lengths in the same units as those of the phylogenetic distances used to infer the phylogenetic tree. (*C*) TMEM43 amino acid sequences of *pogona vitticeps, beluga whale, canis lupus familiaris, bos taurus, homo sapiens, mus musculus, rattus norvegicus, danio rerio* and *astyanax mexicanus* were aligned using 'Clustal Omega' server. The transmembrane (TM) structure with high hydrophobicity for each sequence is represented in orange, intramembrane (IM) domain in blue and ANSD causing p.(Arg372Ter) site is marked with yellow box. Epitope for antibody (Ab) used in this study is marked with gray box.

Fig. S4.

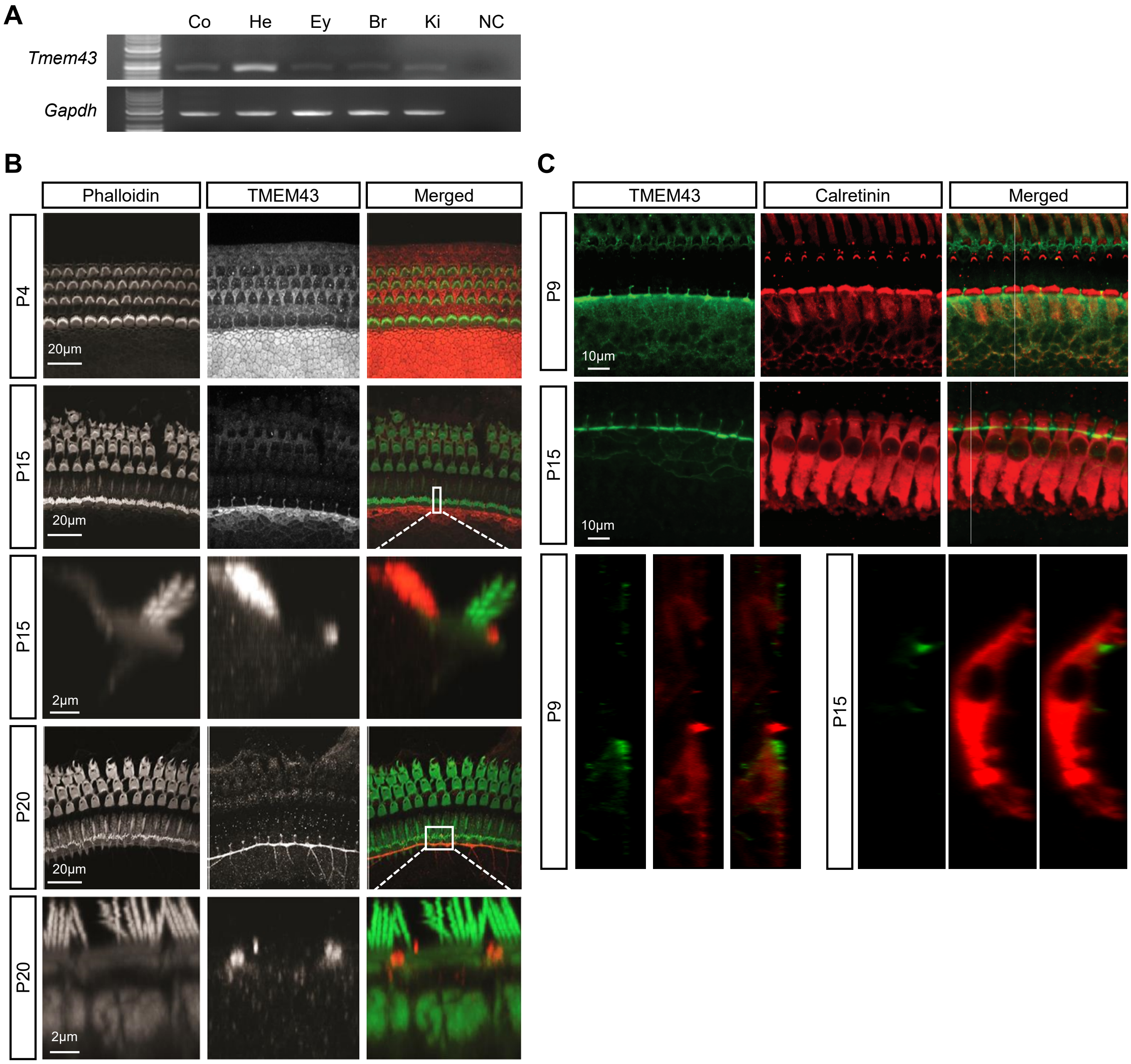
A
Co
He
Ey
Br
Ki
NC
Tmem43
Gapdh
B
Phalloidin
TMEM43
Merged
P4
20µm
P15
20µm
P15
2µm
P20
20µm
P20
2µm
C
TMEM43
Calretinin
Merged
P9
10µm
P15
10µm
P9
P15

Figure S4. TMEM43 expression becomes restricted at inner border cells and cell junctions of the inner sulcus cells.

(*A*) RT-PCR gel analysis of *Tmem43* in various organs of mouse. Single bands at *Tmem43* expression (size 196bp) are shown. Co; cochlea, He; heart, Ey; eye, Br; brain, Ki; kidney, NC; negative control. (*B*) Spatiotemporal expression of TMEM43 (red) and phalloidin (green) at the mouse organ of Corti. TMEM43 immunoreactivity detected widely in the Kolliker's organ at P4 becomes restricted in the course of postnatal development at inner border cells and cell junctions of the inner sulcus cells at P20. Boxed area indicates magnified view of TMEM43 localization at the apical membrane or cortex of the inner border cell. (*C*) Confocal micrographs of cochlear turns with anti-TMEM43 (green) and anti-calretinin (red). Vertical line indicates magnified ZY view in the lower panel. TMEM43 is found outside of calretinin-positive IHC.

Fig. S5.

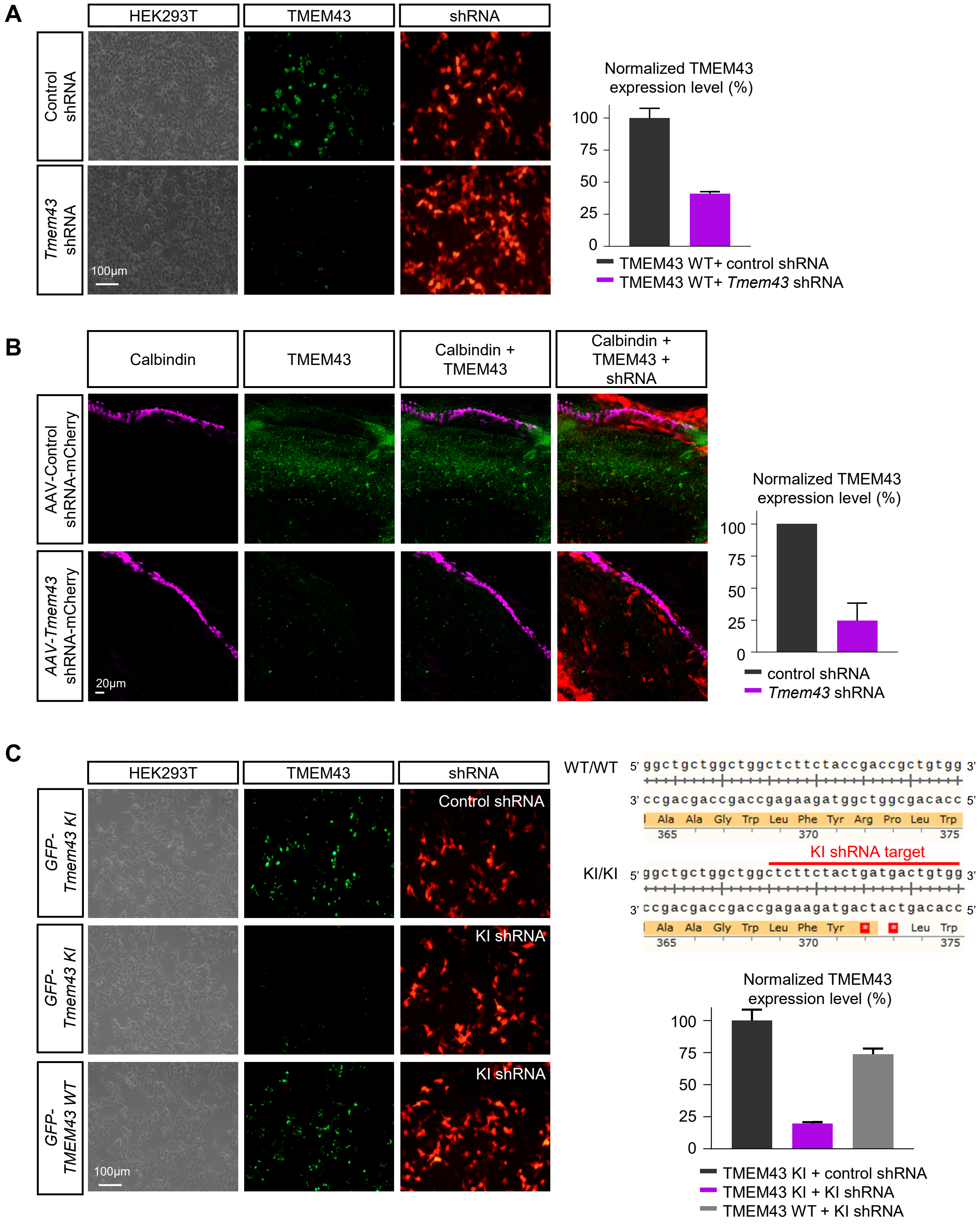
A
HEK293T
TMEM43
shRNA
Control shRNA
Tmem43 shRNA
100µm
Normalized TMEM43 expression level (%)
100
75
50
25
0
TMEM43 WT+ control shRNA
TMEM43 WT+ Tmem43 shRNA
B
Calbindin
TMEM43
Calbindin + TMEM43
Calbindin + TMEM43 + shRNA
AAV-Control shRNA-mCherry
AAV-Tmem43 shRNA-mCherry
20µm
Normalized TMEM43 expression level (%)
100
75
50
25
0
control shRNA
Tmem43 shRNA
C
HEK293T
TMEM43
shRNA
GFP-Tmem43 KI
GFP-Tmem43 KI
GFP-TMEM43 WT
Control shRNA
KI shRNA
KI shRNA
100µm
WT/WT
5' ggctgctggctggctcttctaccgaccgctgtgg 3'
3' ccgacgaccgaccgagaagatggctggcgacacc 5'
Ala Ala Gly Trp Leu Phe Tyr Arg Pro Leu Trp
365 370 375
KI shRNA target
KI/KI
5' ggctgctggctggctcttctactgatgactgtgg 3'
3' ccgacgaccgaccgagaagatgactactgacacc 5'
Ala Ala Gly Trp Leu Phe Tyr Leu Trp
365 370 375
Normalized TMEM43 expression level (%)
100
75
50
25
0
TMEM43 KI + control shRNA
TMEM43 KI + KI shRNA
TMEM43 WT + KI shRNA

Figure S5. m*Tmem43* shRNA effectively targets *Tmem43* in both *in vitro* and *ex vivo* system and *Tmem43* KI shRNA targets KI sequence without interfering with wildtype *Tmem43*.
(*A*) GFP-*Tmem43* transfected with control and candidate *Tmem43* shRNA. *Tmem43* shRNA suppressed TMEM43 expression by average 59%. (*B*) Genetic knockdown of TMEM43 with *Tmem43* shRNA virus on cultured mouse cochlea. IHCs are marked with Calbindin. *Tmem43* shRNA treated cochlea display average 71.4% reduction in TMEM43 signals at GLSs. (*C*) *Tmem43*^{KI} insert sequence targeting shRNA efficiency was tested by co-expression of GFP-*Tmem43* or GFP-*Tmem43 KI* with control shRNA or *Tmem43 KI* shRNA on HEK293T. KI shRNA effectively knocked-down *Tmem43* KI DNA (81%) but was ineffective on *Tmem43* WT DNA (26%). Quantification was measured from each cell (n is 94, 79 and 124 for each group). The *Tmem43* KI DNA and KI targeting shRNA sequence are indicated on right, top. Note that KI targeting shRNA cannot target WT *Tmem43* sequence.

Fig. S6.

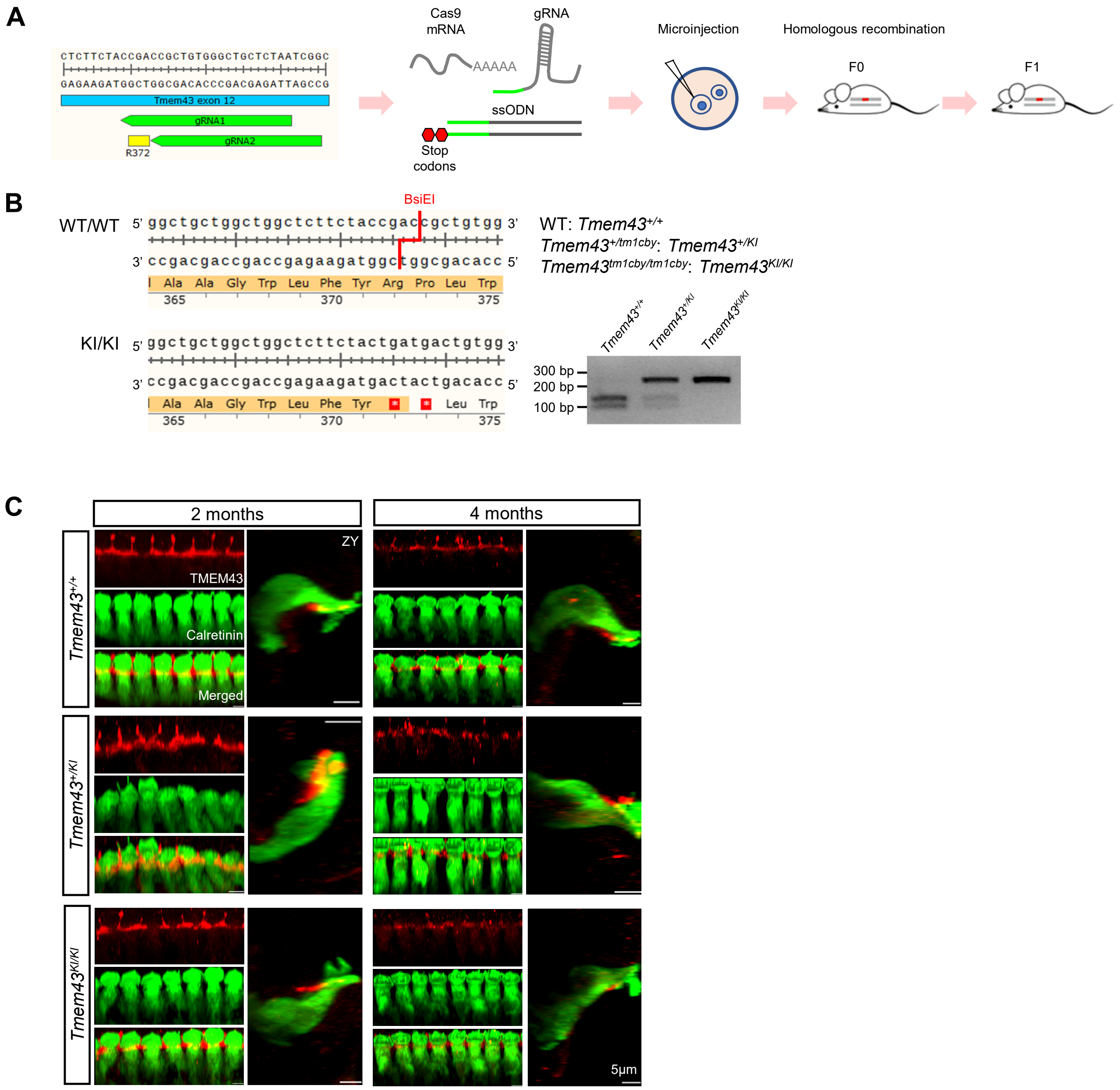

A
CTCTTCTACCGACCGCTGTGGGCTGCTCTAATCGGC
GAGAAGATGGCTGGCGACACCCGACGAGATTAGCCG
Tmem43 exon 12
gRNA1
gRNA2
R372
Cas9 mRNA
AAAAA
gRNA
ssODN
Stop codons
Microinjection
Homologous recombination
F0
F1
B
BsiEI
WT/WT
5' ggctgctggctggctcttctaccgaccgctgtgg 3'
3' ccgacgaccgaccgagaagatggctggcgacacc 5'
Ala Ala Gly Trp Leu Phe Tyr Arg Pro Leu Trp
365 370 375
KI/KI
5' ggctgctggctggctcttctactgatgactgtgg 3'
3' ccgacgaccgaccgagaagatgactactgacacc 5'
Ala Ala Gly Trp Leu Phe Tyr Leu Trp
365 370 375
WT: Tmem43+/+
Tmem43+/tm1cby: Tmem43+/KI
Tmem43tm1cby/tm1cby: Tmem43KI/KI
Tmem43+/+
Tmem43+/KI
Tmem43KI/KI
300 bp
200 bp
100 bp
C
2 months
4 months
ZY
TMEM43
Calretinin
Merged
Tmem43+/+
Tmem43+/KI
Tmem43KI/KI
5μm

Figure S6. Generation of *Tmem43*^{KI} mice and TMEM43 expression in *Tmem43*^{KI} mice.

(*A*) Schematic flow illustrating the procedure in generation of *Tmem43*-p.(Arg372Ter) KI (C57BL/6J;129S-*Tmem43*^{tm1Cby}) mouse model using Clustered Regularly Interspaced Short Palindromic Repeats (CRISPR) technology. Double stop codons were inserted at the region corresponding p.(Arg372). A germline transmission of p.(Arg372Ter) was confirmed. gRNA; guide RNA, ssODN; Single-stranded oligodeoxynucleotide donor. (*B*) The p.(Arg372Ter) site that have been replaced with two stop codons and restriction enzyme site of BsiEI are indicated. Genotyping result using BsiEI is shown. (*C*) Confocal micrographs of the organs of Corti (apical turn) from *Tmem43*$^{+/+}$, *Tmem43*$^{+/KI}$ and *Tmem43*$^{KI/KI}$ mice. TMEM43 immunoreactivity is detected at GLSs of all genotypes. Note compact and restricted TMEM43 in *Tmem43*$^{+/+}$ mice and a dispersed signal in *Tmem43*$^{+/KI}$ and *Tmem43*$^{KI/KI}$ mice.

Fig.S7.

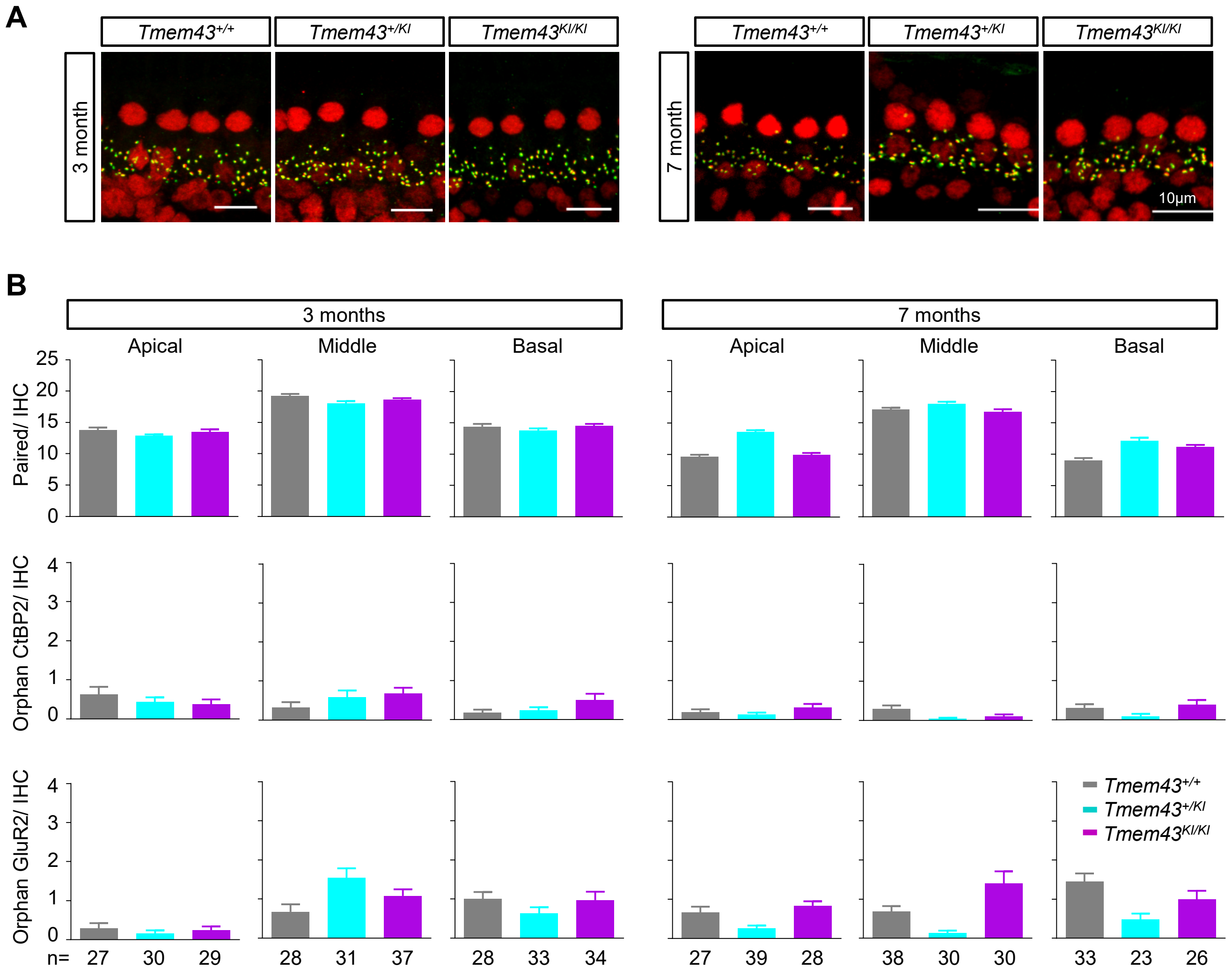

A
Tmem43+/+
Tmem43+/KI
Tmem43KI/KI
3 month
7 month
10μm
B
3 months
7 months
Apical
Middle
Basal
Paired/ IHC
Orphan CtBP2/ IHC
Orphan GluR2/ IHC
Tmem43+/+
Tmem43+/KI
Tmem43KI/KI
n= 27 30 29 28 31 37 28 33 34 27 39 28 38 30 30 33 23 26

Figure S7. *Tmem43*^{KI} mice display normal ribbon synapses.

(A) Immunofluorescence staining for CtBP2/RIBEYE (red) and GluA2 (green) to visualize IHC ribbon synapses in 3- and 7-month-old *Tmem43*$^{+/+}$, *Tmem43*$^{+/KI}$ and *Tmem4*$^{KI/KI}$ mice. Most CtBP2 puncta juxtapose GluA2 puncta. scale bar = 10 μm. (B) Number of paired CtBP2 and GluA2 puncta (within 1μm from each other) and orphan CtBP2, orphan GluA2 puncta per IHC (mean ± SEM) in the apical (segment corresponding to 0-25 % from the apex), middle (40-60 % from apex) and basal area (> 75% from apex) of cochlea from *Tmem43*$^{+/+}$, *Tmem43*$^{+/KI}$ and *Tmem4*$^{KI/KI}$ mice. n represents number of IHCs analyzed in each group (2 animals, respectively). At all ages and cochlear regions examined, there was no statistical difference in the number CtBP2 puncta between genotypes.

Fig.S8

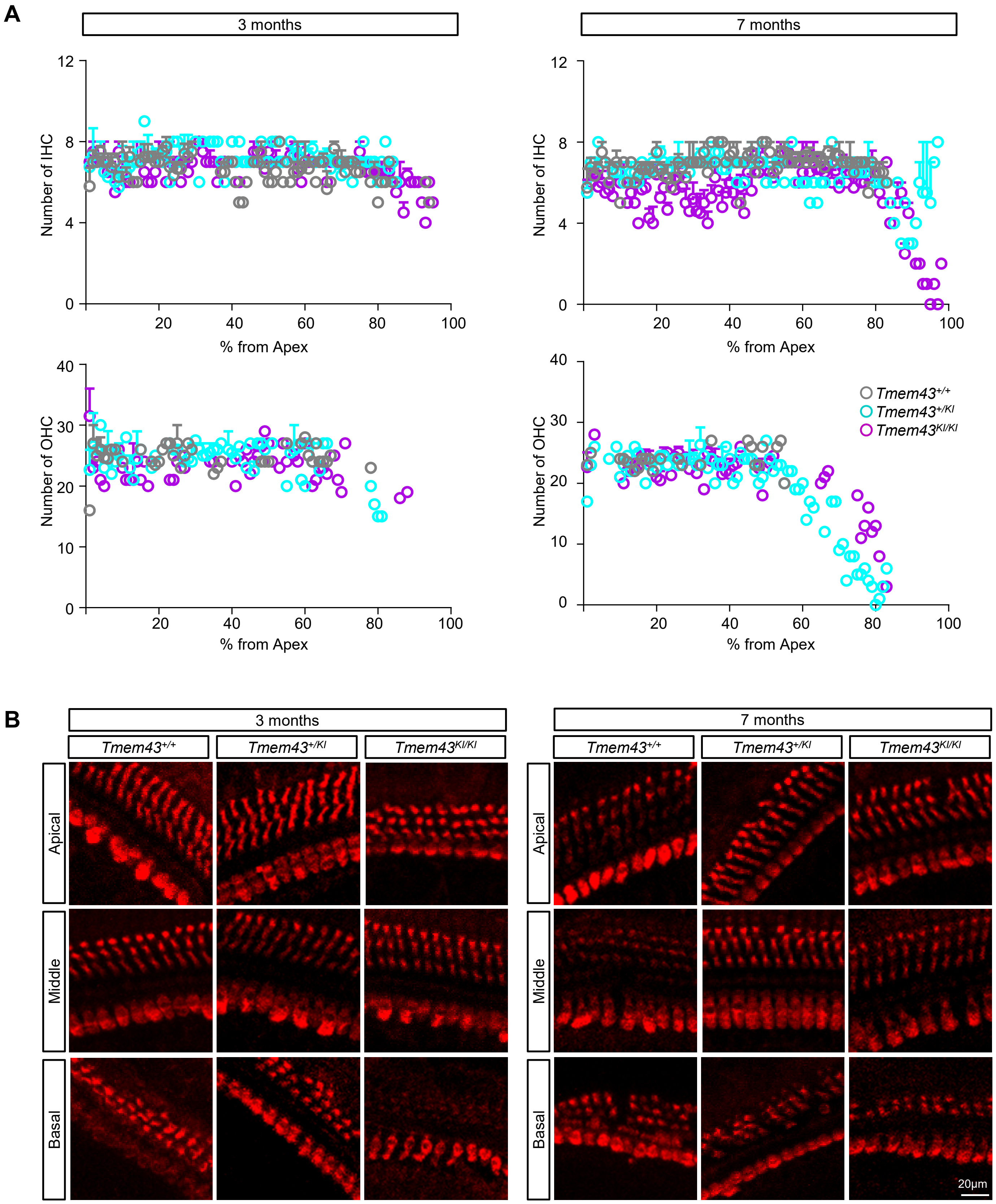

A
3 months
7 months
Number of IHC
12
8
4
0
20
40
60
80
100
% from Apex
Number of OHC
40
30
20
10
Tmem43+/+
Tmem43+/KI
Tmem43KI/KI
B
3 months
7 months
Tmem43+/+
Tmem43+/KI
Tmem43KI/KI
Apical
Middle
Basal
20μm

Figure S8. Number of hair cell comparison between *Tmem43*$^{+/+}$, *Tmem43*$^{+/KI}$, and *Tmem43*$^{KI/KI}$ mouse cochlea.

(*A*) Number of hair cells in segment spanning 1% of the whole cochlear length in 3- and 7-month-old *Tmem43*$^{+/+}$, *Tmem43*$^{+/KI}$ and *Tmem4*$^{KI/KI}$ mice. Neither IHC nor OHC number shows significant difference in 3-month-old mice. A slight decrease in the number of IHC (approximately 15-45% segment) was observed in 7-month-old *Tmem4*$^{KI/KI}$. (n=5, 4, and 4 cochleae for IHC analysis in 3 month-old; n=4, 4, and 4 cochleae for OHC analysis in 3-month old; n=4, 4, 5 cochleae for IHC analysis in 7-month old; n=3, 3, 5 cochleae for OHC analysis in 7-month old mice, respectively) (*B*) Representative images of hair cells in apical (15-20% from the apex), middle (50-55% from the apex), and basal (68-85% from the apex) turns. IHCs and OHCs were immunolabeled with anti-myosin 6.

Fig.S9

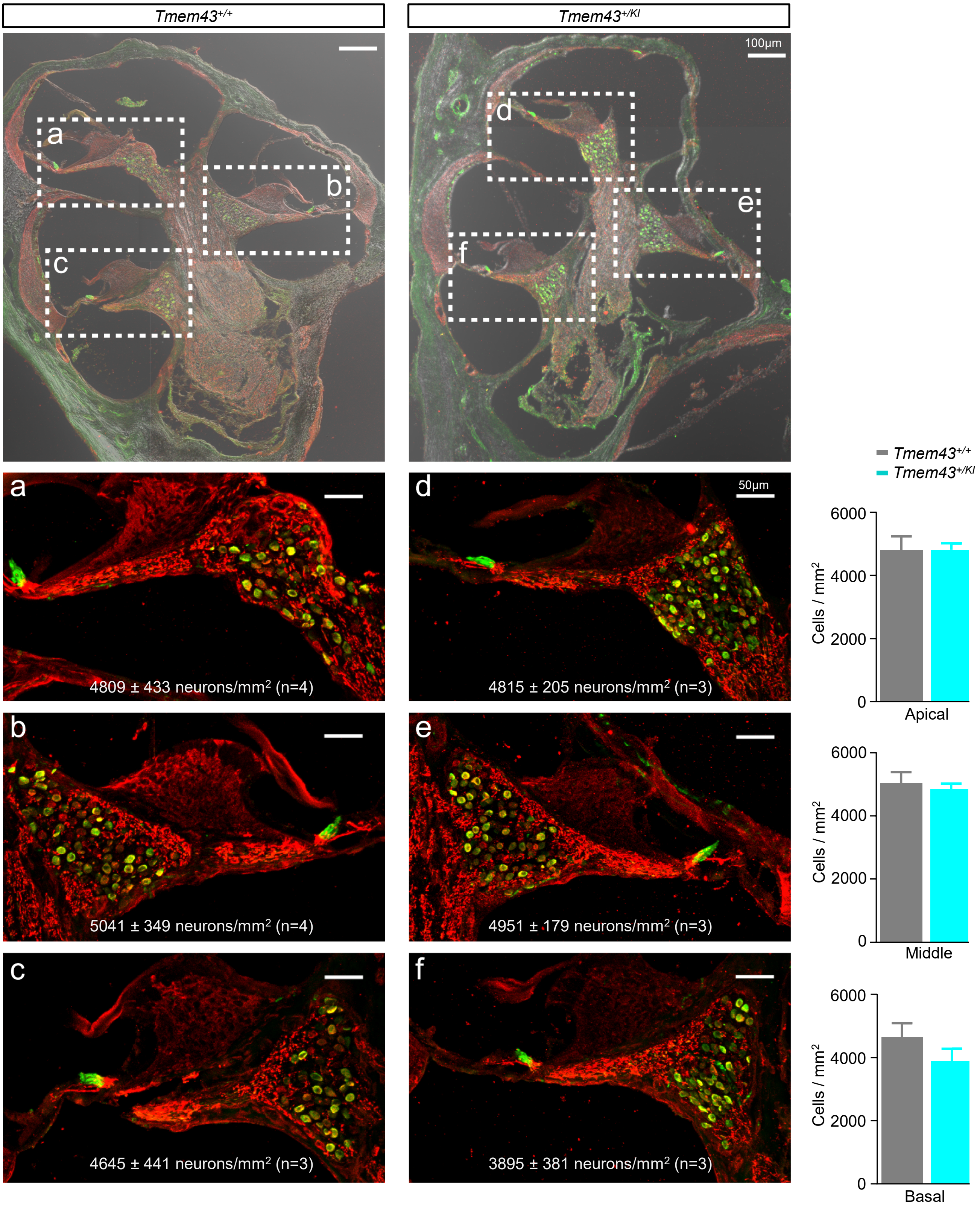

Tmem43+/+
Tmem43+/KI
100µm
a
b
c
d
e
f
50µm
4809 ± 433 neurons/mm² (n=4)
4815 ± 205 neurons/mm² (n=3)
5041 ± 349 neurons/mm² (n=4)
4951 ± 179 neurons/mm² (n=3)
4645 ± 441 neurons/mm² (n=3)
3895 ± 381 neurons/mm² (n=3)
Tmem43+/+
Tmem43+/KI
Cells / mm²
6000
4000
2000
0
Apical
Middle
Basal

Figure S9. SGN density in 6-month-old *Tmem43*^{KI} mice.
Frozen-section images of cochleae from 6-month-old *Tmem43*$^{+/+}$ (left) and *Tmem43*$^{+/KI}$ (right) mice. Frozen cochlear sections were immunolabeled with anti-calretinin (green) and anti-neurofilament H (red). scale bar = 100μm. Boxed areas indicate magnified views in lower panels, each corresponding to the apical (a, d), middle (b, e), and basal area (c, f) of cochlea. Data are presented as mean ± SD. n indicates the number of cochleae. 2-4 frozen sections per cochlea were analyzed. scale bar = 50μm. At all cochlear regions examined, there was no statistical difference in the SGN density between genotypes.

Fig. S10.

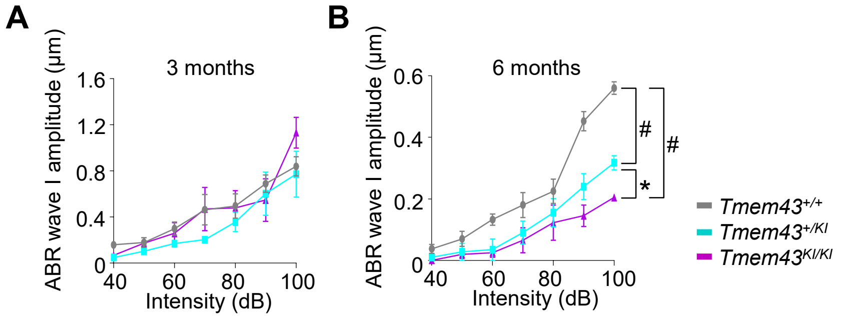
A
3 months
ABR wave I amplitude (μm)
1.6
1.2
0.8
0.4
0
40
60
80
100
Intensity (dB)
B
6 months
ABR wave I amplitude (μm)
0.6
0.4
0.2
0
40
60
80
100
Intensity (dB)
#
#
*
Tmem43+/+
Tmem43+/KI
Tmem43KI/KI

Figure S10. Growth function curve of suprathreshold ABR Wave I amplitude from *Tmem43*^{KI} mice at 3 and 6 months.

(*A*) Mean ± SEM ABR wave I amplitude growth function for *Tmem43*$^{+/+}$ (gray, n=3), *Tmem43*$^{+/KI}$ (cyan, n=3) and *Tmem43*$^{KI/KI}$ (purple, n=3) with response to 8kHz tone burst sound at 3 months after birth. ABR wave I amplitudes are not significantly different among each genotype at whole decibels measured up to 3 months except at 70dB. (*B*) Mean ± SEM ABR wave I amplitude growth function for littermate *Tmem43*$^{+/+}$ (gray, n=4), *Tmem43*$^{+/KI}$ (cyan, n=4) and *Tmem43*$^{KI/KI}$ (purple, n=4) at 8kHz measured at 6 months after birth. Wave I amplitudes of *Tmem43*$^{+/KI}$ and *Tmem43*$^{KI/KI}$ mice are significantly reduced over several intensities compared with *Tmem43*$^{+/+}$ with response to 8kHz tone burst sound where there is no difference in ABR thresholds among genotypes.

Fig. S11.

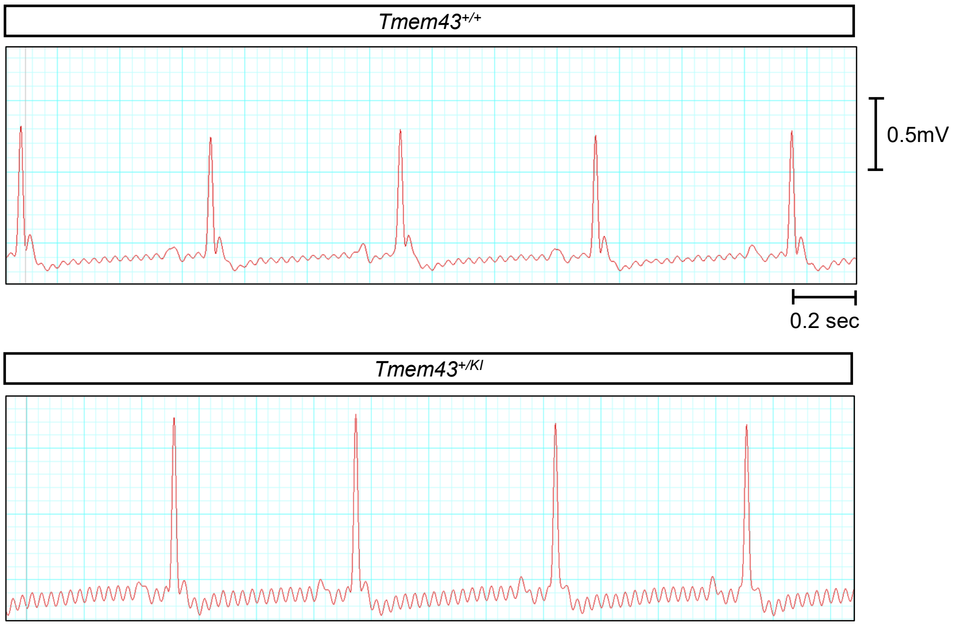

Figure S11. *Tmem43*^{KI} mice display no symptoms of ARVC.
Representative electrocardiography (ECG) results from littermates of *Tmem43*$^{+/+}$ and *Tmem43*$^{+/KI}$ mice. It was measured at 6 months after birth, showing that *Tmem43*^{KI} mice display normal heart function.

Fig. S12

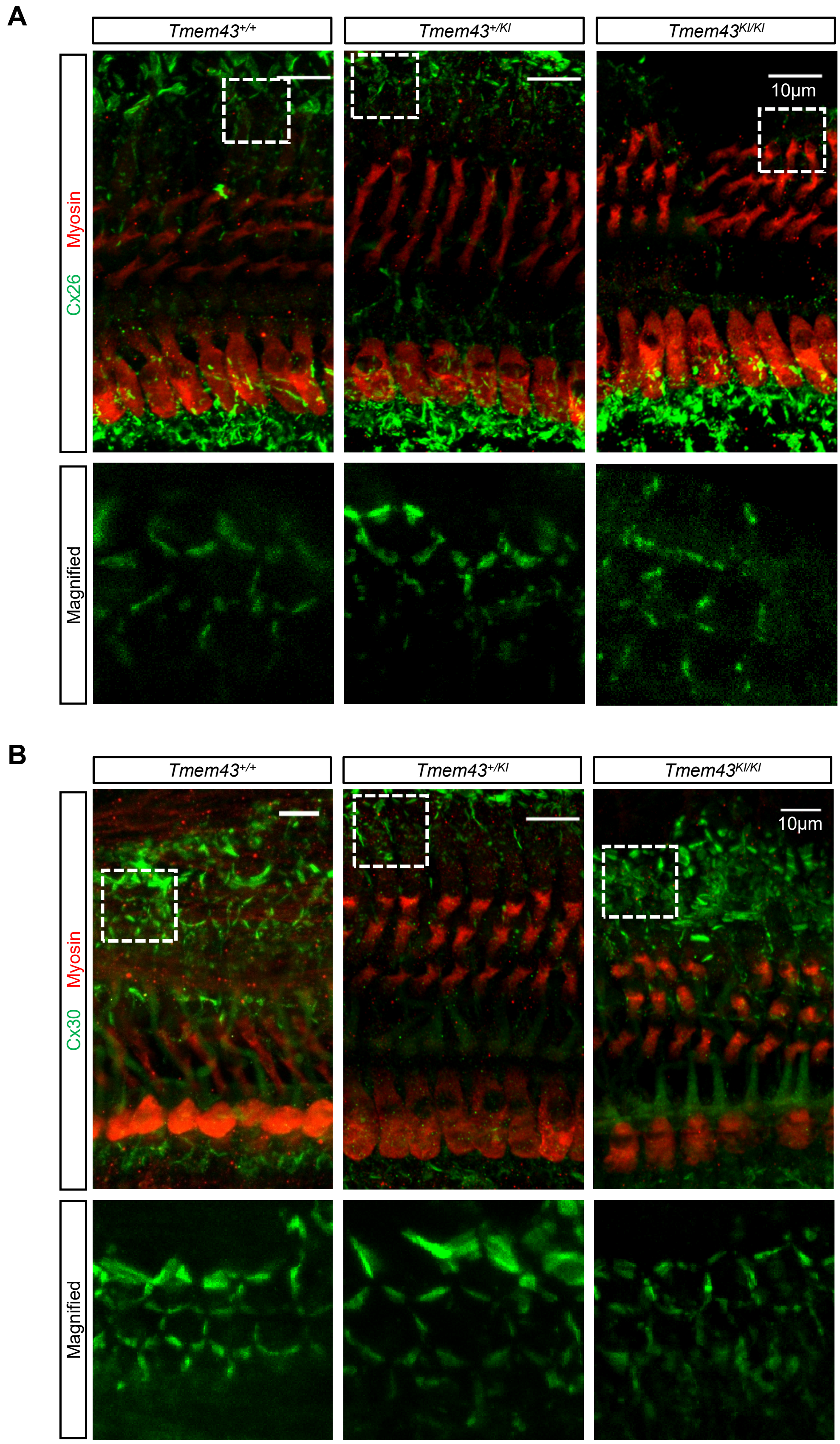

A
Tmem43+/+
Tmem43+/KI
Tmem43KI/KI
10μm
Cx26 Myosin
Magnified
B
Tmem43+/+
Tmem43+/KI
Tmem43KI/KI
10μm
Cx30 Myosin
Magnified

Figure S12. Cx26 and Cx30 expression in the *Tmem43*$^{+/+}$, *Tmem43*$^{+/KI}$ and *Tmem43*$^{KI/KI}$ mouse cochlea.
(*A*) Confocal micrographs of Cx26 (green) and myosin 6 (red) expression in the organ of Corti of *Tmem43*$^{+/+}$, *Tmem43*$^{+/KI}$ and *Tmem43*$^{KI/KI}$ mice (5 month). (*B*) Confocal micrographs of Cx30 (green) and myosin 6 (red) expression in the organ of Corti of *Tmem43*$^{+/+}$, *Tmem43*$^{+/KI}$ and *Tmem43*$^{KI/KI}$ mice (7 month). Boxed area indicates magnified view of outer sulcus in lower panel. There was no difference among the genotypes.

Fig. S13.

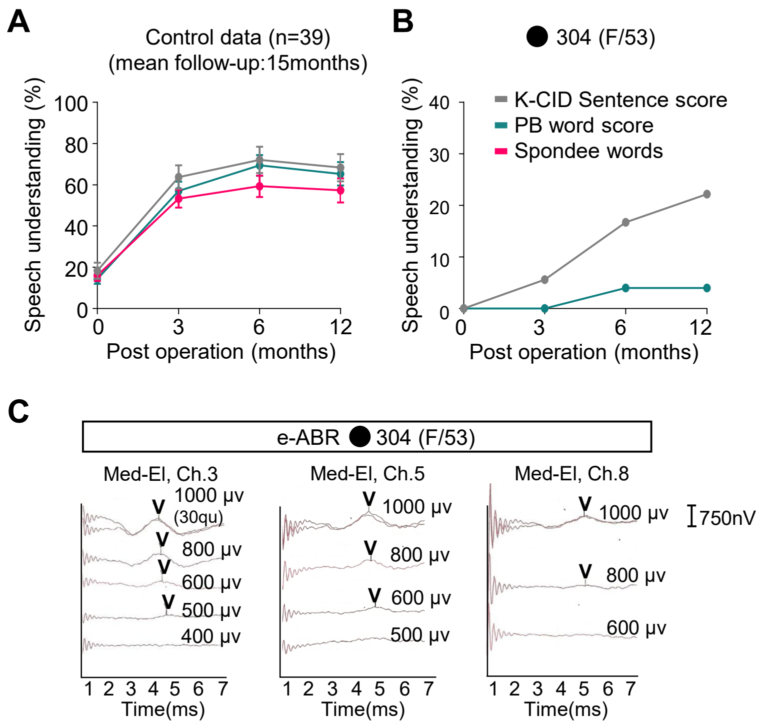

A
Control data (n=39)
(mean follow-up:15months)
Speech understanding (%)
100
80
60
40
20
0
0
3
6
12
Post operation (months)
B
304 (F/53)
Speech understanding (%)
40
30
20
10
0
K-CID Sentence score
PB word score
Spondee words
Post operation (months)
C
e-ABR 304 (F/53)
Med-El, Ch.3
1000 µv
(30qu)
800 µv
600 µv
500 µv
400 µv
Med-El, Ch.5
1000 µv
800 µv
600 µv
500 µv
Med-El, Ch.8
1000 µv
800 µv
600 µv
750nV
V
1 2 3 4 5 6 7
Time(ms)

Figure S13. Prolonged deafness duration reduces restoration of speech discrimination ability after cochlea implantations, presumably due to cortical damage.

(*A*) Longitudinal change of postoperative auditory performance from control subjects (n=39) with adult onset progressive hearing loss: Scores at postoperative 3, 6, and 12 months are shown. (*B*) Subject #304 showed improved K-CID (up to 22.2%) and PB word (up to 4%) score after cochlear implantation. (*C*) The responses of electrical auditory brainstem responses (e-ABR) obtained by cochlear implanted electrodes inserted in the right ear (shown in red) of subject #304. From the channels tested (No. 3, 5, 8), the wave V (annotated on the waves) was detected at about 4ms after electrical stimulation and the thresholds ranged from 500 to 800μV,indicative of responsiveness of cochlear nerve to stimuli after cochlea implantation. K-CID Sentence score; Korean Central Institute for the Deaf score, PB word score; phonetically balanced word score, Spondee words; audiology a word with 2 syllables–disyllabic, that is pronounced with equal emphasis the 1st and 2nd syllables.

Fig. S14.

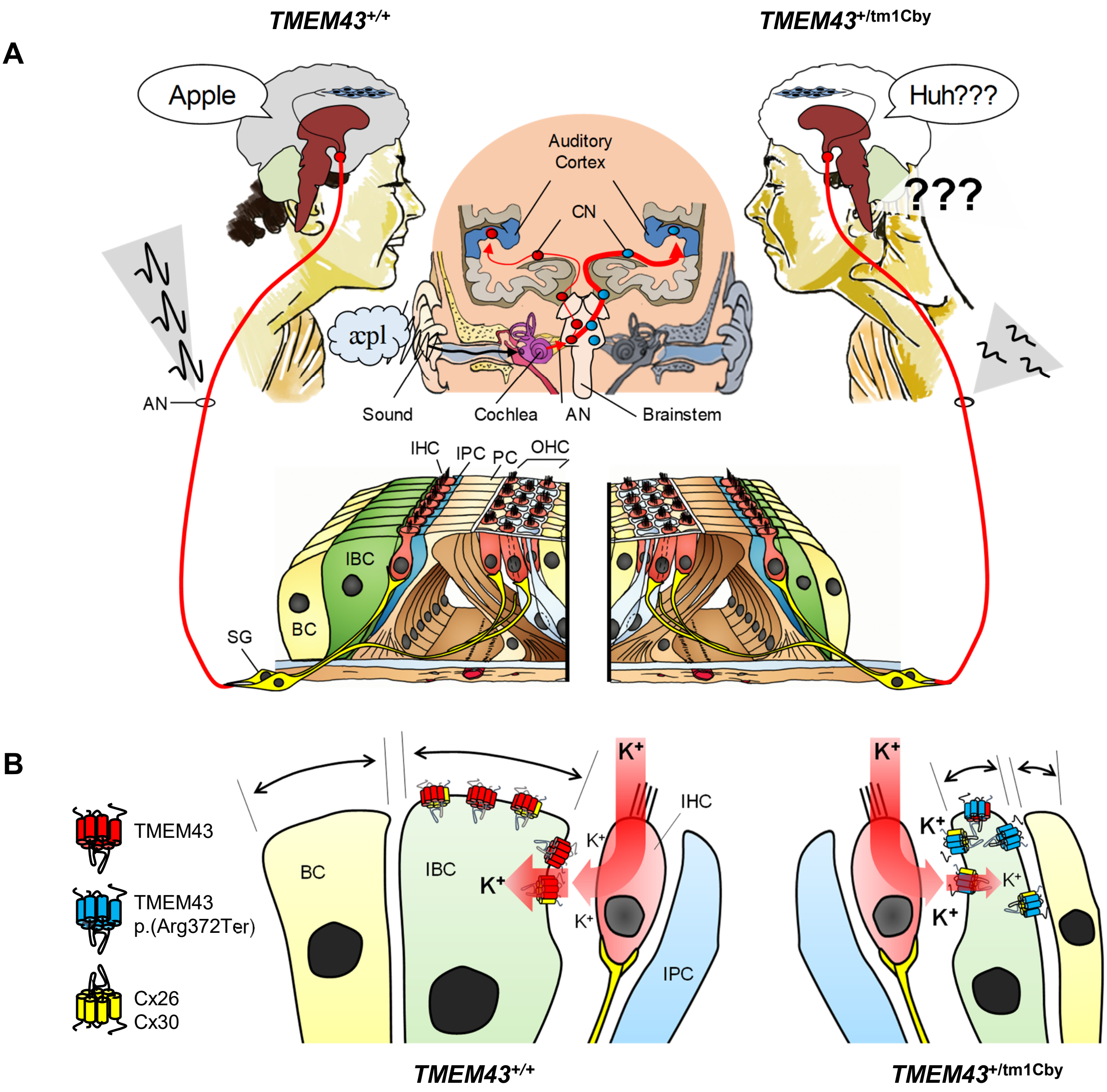

A
TMEM43+/+
TMEM43+/tm1Cby
Apple
Huh???
???
Auditory Cortex
CN
Sound
Cochlea
AN
Brainstem
AN
IHC
IPC
PC
OHC
IBC
BC
SG
B
TMEM43
TMEM43 p.(Arg372Ter)
Cx26
Cx30
BC
IBC
K+
IHC
IPC
TMEM43+/+
TMEM43+/tm1Cby

Figure S14. Schematic diagram illustrating the role of TMEM43 in the cochlear GLS.

(*A*) Subjects carrying a p.(Arg372Ter) of *TMEM43* (right) demonstrate significantly deteriorated speech discrimination compared with normal subjects (left), which is attributed to the functional and morphological abnormalities of GLSs. (*B*) Schematic diagram for the expression of TMEM43 and connexins in the cochlear GLSs. The cochlear GLSs with TMEM43-p.(Arg372Ter) (right) display significantly decreased K^+-mediated passive conductance current compared to its WT counterpart (left) and its capability of maintaining the cellular volume is disturbed. IHC; Inner hair cell, OHC; Outer hair cell, IBC; Inner border cell, BC; Border cell, AN; Auditory nerve, CN; Cochlear nucleus, SG; Spiral ganglion.

Table S1. Nonsynonymous variants within the locus* co-segregating with ANSD that survived filtering process after exome sequencing among 3 subjects from HN66

Func	Gene	ExonicFunc	GenbankID	Position	Exon	Nucleotide	AA	Chr	Start	End	Ref	Alt	Global MAF	KRGDB (N=1722)	PolyPhen-2	SIFT	GERF++
**Exome sequencing**																	
exonic	**TMEM43**	stopgain SNV	NM_024334	Region # 1** (3p25.1)	exon12	c.C1114T	p.R372X	chr3	14183206	14183206	C	T	T=0.000008/1 (ExAC)	ND	NA	NA	4.83
exonic	XIRP1	nonsynonymous SNV	NM_001198621		exon2	c.T281C	p.M94T	chr3	39230656	39230656	A	G	G=0.0000165/2 (ExAC)	ND	0.998	0.784	4.93
exonic	VILL	frameshift deletion	NM_015873		exon9	c.946delT	p.F316fs	chr3	38040406	38040406	T	-	0.0001(ExAC)	ND	NA	NA	NA
exonic	CTBP2	nonsynonymous SNV	NM_022802		exon5	c.T2315G	p.L772W	chr10	126683123	126683123	A	C	NA	ND	1	0.912	5.21
exonic	CHODL	nonsynonymous SNV	NM_ 024944		exon5	c.T665C	p.I222T	chr21	19635138	19635138	T	C	C= 0.000008/1 (ExAC)	ND	0.999	0.721	5.39

* Region #1: Chr 3: 13,165,401-22,769,511; Ref: Reference sequence; Alt: Alternate sequence; ND: not detected; NA: not applicable.

The candidate variants were firstly listed up if they were nonsynonymous variants in coding regions. Variants with a minor allele frequency less than 1% were then selected based on the Exome Sequencing Project 6500 (ESP6500), the 1000 Genome Project (1000G), The Exome Aggregation Consortium (ExAC), Trans-Omics for Precision Medicine (TOPMED) and our in-house database containing the exomes of 81 Korean individuals. Following the inheritance pattern, homozygous variants and compound heterozygote variants with sufficient read depths (>10X) and a genotype quality (>20) were selected commonly found in the affected siblings. Finally, the variants which have none of clinical significance of dbSNP ID were finally identified. In silico prediction Algorithm: Polyphen-2 (9); SIFT (10, 11); Conservation tools: GERP++ score in the UCSC Genome Browser (12); ExAC, Exome Aggregation Consortium (13); 1000 Genomes (14); KRGDB, Korean Reference Genome DB (15); TOPMED, Trans-Omics for Precision Medicine (16).

Table S2. Nonsynonymous variants within the locus* co-segregating with ANSD that survived filtering process after exome sequencing among 4 subjects from SB162

Func	Gene	ExonicFunc	GenbankID	Position	Exon	Nucleotide	AA	Chr	Start	End	Ref	Alt	Global MAF	KRGDB (N=1722)	PolyPhen-2	SIFT	GERF++
**Targeted exome sequencing after linkage analysis**																	
exonic	**TMEM43**	Stopgain SNV	NM_024334	Region # 2** (3p25.1)	exon12	c.C1114T	p.R372X	chr3	14,183,206	14,183,206	C	T	T=0.000008/1 (ExAC)	ND	NA	NA	4.83
exonic	FBLN2	nonsynonymous SNV	NM_001998	Region # 2** (3p25.1)	exon11	c.G2551C	p.D851H	chr3	13,670,527	13,670,527	G	C	C=0.0007/84 (ExAC) C=0.0004/2 (1000 Genomes)	C=0.005517/19	0.893	0.04	4.23
**Exome sequencing**																	
exonic	KIAA1614	nonsynonymous SNV	NM_020950		exon5	c.G2514T	p.E838D	chr1	180905559	180905559	G	T	ND	ND	0.002	0.39	-5.05
exonic	ADCK3	nonsynonymous SNV	NM_020247		exon6	c.C818T	p.A273V	chr1	227169815	227169815	C	T	T=0.00006/5 (ExAC) T=0.00008/1 (GO-ESP) T=0.0001/4 (TOPMED)	ND	0.999	0.04	5.1
exonic	TBCE	nonsynonymous SNV	NM_001079515		exon9	c.T794C	p.I265T	chr1	235599116	235599116	T	C	ND	ND	0.01	0.62	4.61
exonic	PARD3B	nonsynonymous SNV	NM_057177		exon11	c.A1594C	p.I532L	chr2	206023605	206023605	A	C	ND	ND	0.28	0.08	-1.97
exonic	HDLBP	nonsynonymous SNV	NM_001243900		exon21	c.G2728A	p.G910S	chr2	242176107	242176107	C	T	T=0.000008/1 (ExAC) T=0.0002/1 (1000 Genomes)	ND	0.094	0.64	0.666
exonic	**TMEM43**	stopgain SNV	NM_024334		exon12	c.C1114T	p.R372X	chr3	14183206	14183206	C	T	T=0.000008/1 (ExAC)	ND	NA	NA	4.83
exonic	DTX3L	nonsynonymous SNV	NM_138287		exon1	c.G83A	p.S28N	chr3	122283356	122283356	G	A	ND	ND	0.454	0.03	-9.39
exonic	KIAA1024L	nonsynonymous SNV	NM_001257308		exon1	c.T160G	p.S54A	chr5	129084043	129084043	T	G	ND	ND	NA	NA	3.53
exonic	P4HA2	nonsynonymous SNV	NM_001017973		exon2	c.T80C	p.I27T	chr5	131554240	131554240	A	G	ND	G=0.00029/1	0.01	0.07	6.17
exonic	DOK3	nonsynonymous SNV	NM_001144875		exon2	c.C8T	p.P3L	chr5	176936534	176936534	G	A	ND	ND	0.991	0.03	4.47
exonic	MAML1	nonsynonymous SNV	NM_014757		exon5	c.C2338T	p.H780Y	chr5	179201165	179201165	C	T	ND	T=0.00029/1	0.916	0.31	4.94
exonic	ARID1B	nonframeshift insertion	NM_017519		exon1,exon1	c.1029_1030insGCG	p.A343delinsAA	chr6	157100092	157100092	-	GCG	ND	InsGCG=0.001742/6	NA	NA	-0..657
exonic	EPC1	nonsynonymous SNV	NM_001272004		exon4	c.T572C	p.I191T	chr10	32582010	32582010	A	G	ND	ND	0.999	0.07	6.04
exonic	CRYAB	nonsynonymous SNV	NM_001885		exon1	c.T79A	p.F27I	chr11	111782370	111782370	A	T	ND	ND	0.002	0.35	2.56
exonic	INO80	nonsynonymous SNV	NM_017553		exon6	c.A623G	p.K208R	chr15	41379795	41379795	T	C	C=0.0001/17 (ExAC)	C=0.0046457/16	0.009	0.61	4.2
exonic	MYO5C	nonsynonymous SNV	NM_018728		exon29	c.A3620C	p.E1207A	chr15	52515748	52515748	T	G	ND	ND	0.365	0.73	5.97
exonic	CILP	nonsynonymous SNV	NM_003613		exon5	c.C448G	p.R150G	chr15	65497781	65497781	G	C	ND	ND	0.0	0.67	0.901
exonic	MAP2K1	nonsynonymous SNV	NM_002755		exon10	c.A1062C	p.Q354H	chr15	66782095	66782095	A	C	C=0.00002/2 (ExAC)	C=0.001161/4	0.001	0.02	-5.39
exonic	ZFPM1	nonsynonymous SNV	NM_153813		exon10	c.C1645G	p.L549V	chr16	88600011	88600011	C	G	ND	G=0.00029/1	0.978	0.01	2.61
exonic	MIDN	nonsynonymous SNV	NM_177401		exon5	c.C401T	p.S134L	chr19	1254182	1254182	C	T	T=0.00003/2 (ExAC) T=0.0002/5 (TOPMED)	ND	1	0	3.8

* Locus: Region 1+ Region 2 (See Figure 1c inset); ** Region #1: Chr 3: 1,946,000 - 5,956,000; Region #2: Chr 3:11,883,000-14,502,000; Ref: Reference sequence; Alt: Alternate sequence; ND: not detected; NA: not applicable. The candidate variants were firstly listed up if they were nonsynonymous variants in coding regions. Variants with a minor allele frequency less than 1% were then selected based on the Exome Sequencing Project 6500 (ESP6500), the 1000 Genome Project (1000G), The Exome Aggregation Consortium (ExAC), Trans-Omics for Precision Medicine (TOPMED) and our in-house database containing the exomes of 81 Korean individuals. Following the inheritance pattern, homozygous variants and compound heterozygote variants with sufficient read depths (>10X) and a genotype quality (>20) were selected commonly found in the affected siblings. Finally, the variants which have none of clinical significance of dbSNP ID were finally identified. In silico prediction Algorithm: Polyphen-2 (9); SIFT (10, 11); Conservation tools: GERP++ score in the UCSC Genome Browser (12); ExAC, Exome Aggregation Consortium (13); 1000 Genomes (14); KRGDB, Korean Reference Genome DB (15); TOPMED, Trans-Omics for Precision Medicine (16).

Table S3. Non-coding region variants within region#2 that survived filtering process after whole genome sequencing among 8 subjects from SB162

Chr	Start	End	Ref	Alt	Func.refGene	Gene.refGene	MAF	Affected group	Unaffected group	Normal Control
chr3	2722187	2722187	G	A	intronic	CNTN4	T=0.0002/1 (1000 Genomes)<br>A=0.000008/1 (TOPMED)<br>A=0.00003230/1 (gnomAD)			●
chr3	3449476	3449476	C	T	intergenic	CRBN,LRRN1	ND			●
chr3	3630012	3630012	G	A	intergenic	CRBN,LRRN1	ND			●
chr3	3765131	3765131	T	-	intergenic	CRBN,LRRN1	-=0.0766/9615 (TOPMED)<br>-= 0.07394/2093 (gnomAD)	X		
chr3	3774063	3774063	T	G	intergenic	CRBN,LRRN1	G=0.00002/3 (TOPMED)<br>G= 0.00003228/1 (gnomAD)			●
chr3	4300751	4300751	A	G	intergenic	LRRN1,SETMAR	ND	X		
chr3	4352459	4352459	-	A	intronic	SETMAR	A=0.0001/15 (TOPMED)			●
chr3	4619624	4619625	AC	-	intronic	ITPR1	-=0.0002259/7 (gnomAD)<br>-=0.0003704/6 (EAS- gnomAD)			
chr3	4619626	4619626	-	TC	intronic	ITPR1	-=0.0002259/7 (gnomAD)<br>-=0.0003704/6 (EAS- gnomAD)			
chr3	5003582	5003582	A	G	ncRNA_intronic	BHLHE40-AS1	ND	X		●
chr3	5452014	5452014	A	G	intergenic	MIR4790,GRM7-AS3	ND			●
chr3	5700196	5700196	C	T	intergenic	MIR4790,GRM7-AS3	T=0.00006459/2 (gnomAD)<br>T=0.001250/2 (EAS- gnomAD)			
chr3	12365259	12365259	-	A	intronic	PPARG	ND		●	
chr3	12531584	12531584	A	G	intronic	TSEN2	ND			●
chr3	13023564	13023564	C	T	intronic	IQSEC1	ND			●
chr3	13229090	13229090	G	A	intergenic	IQSEC1,NUP210	ND			●
chr3	13792436	13792436	T	C	intergenic	LINC00620,WNT7A	ND			●
chr3	14079864	14079864	A	G	ncRNA_intronic	TPRXL	ND			●
chr3	14183206	14183206	C	T	Exonic_stopgain	TMEM43	T=0.000008/1 (ExAC)<br>T=0.000008/1 (TOPMED)<br>T=0.00001 (2/246212, GnomAD)			

X, variants not detected in affected group; ●, variants detected in unaffected group, normal control

Movie S1. TMEM43 expression at GLS of P15 cochlea.

Movie of high magnification confocal 3D micrographs of the mouse cochlea at P15 immunolabeled by a polyclonal anti-TMEM43 (green) counterstained by phalloidin (red) and DAPI staining (blue) shows localization of TMEM43 in the apical membrane or cortex of the inner border cell.

Movie S2. TMEM43 expression at GLS of P20 cochlea is restricted to inner border cells.

Movie of high magnification confocal 3D micrographs of the mouse cochlea at P20 immunolabeled by a polyclonal anti-TMEM43 (green) counterstained by phalloidin (red) and DAPI staining (blue) displays TMEM43 staining more restricted to the plasma membranes of inner border cells and cell junctions of the inner sulcus cells.

References

1. N. K. Kim et al., Whole-exome sequencing reveals diverse modes of inheritance in sporadic mild to moderate sensorineural hearing loss in a pediatric population. Genetics in medicine : official journal of the American College of Medical Genetics 17, 901-911 (2015).

2. S. Sang et al., Proband Whole-Exome Sequencing Identified Genes Responsible for Autosomal Recessive Non-Syndromic Hearing Loss in 33 Chinese Nuclear Families. Frontiers in genetics 10, 639 (2019).

3. O. Akil, L. R. Lustig, Mouse Cochlear Whole Mount Immunofluorescence. Bio Protoc 3 (2013).

4. A. Viberg, B. Canlon, The guide to plotting a cochleogram. Hear. Res. 197, 1-10 (2004).

5. J. O. Shin et al., CTCF Regulates Otic Neurogenesis via Histone Modification in the Neurog1 Locus. Mol. Cells 41, 695-702 (2018).

6. A. Ventura et al., Cre-lox-regulated conditional RNA interference from transgenes. Proceedings of the National Academy of Sciences of the United States of America 101, 10380-10385 (2004).

7. M. A. Kim et al., Methionine Sulfoxide Reductase B3-Targeted In Utero Gene Therapy Rescues Hearing Function in a Mouse Model of Congenital Sensorineural Hearing Loss. Antioxid Redox Signal 24, 590-602 (2016).

8. P. D. Tamura K, Peterson N, Stecher G, Nei M, and Kumar S, MEGA5: Molecular Evolutionary Genetics Analysis using Maximum Likelihood, Evolutionary Distance, and Maximum Parsimony Methods. Molecular Biology and Evolution. (2011).

9. I. A. Adzhubei et al., A method and server for predicting damaging missense mutations. Nat Methods 7, 248-249 (2010).

10. Y. Choi, G. E. Sims, S. Murphy, J. R. Miller, A. P. Chan, Predicting the functional effect of amino acid substitutions and indels. PLoS One 7, e46688 (2012).

11. Y. Choi (2012) A fast computation of pairwise sequence alignment scores between a protein and a set of single-locus variants of another protein. in Proceedings of the ACM

Conference on Bioinformatics, Computational Biology and Biomedicine (Association for Computing Machinery, Orlando, Florida), pp 414–417.

12. W. J. Kent et al., The human genome browser at UCSC. Genome Res. 12, 996-1006 (2002).

13. K. J. Karczewski et al., The mutational constraint spectrum quantified from variation in 141,456 humans. Nature 581, 434-443 (2020).

14. C. Genomes Project et al., A global reference for human genetic variation. Nature 526, 68-74 (2015).

15. Korea National Research Institute of Health. Korean Reference Genome Database (KRGDB). [Updated on April 2018] http://152.99.75.168/KRGDB.

16. J. Hecker et al., A unifying framework for rare variant association testing in family-based designs, including higher criticism approaches, SKATs, and burden tests. Bioinformatics
